# The WRKY transcription factor *GhWRKY27* coordinates the senescence regulatory pathway in upland cotton (*Gossypium hirsutum* L.) 

**DOI:** 10.1186/s12870-019-1688-z

**Published:** 2019-03-29

**Authors:** Lijiao Gu, Lingling Dou, Yaning Guo, Hantao Wang, Libei Li, Congcong Wang, Liang Ma, Hengling Wei, Shuxun Yu

**Affiliations:** State Key Laboratory of Cotton Biology, Cotton Research Institute, Chinese Academy of Agricultural Sciences, Anyang, 455000 China

**Keywords:** ChIP-seq, Cotton, *GhWRKY27*, Leaf senescence, SAGs, Transcription factor

## Abstract

**Background:**

Premature senescence can reduce the yield and quality of crops. WRKY transcription factors (TFs) play important roles during leaf senescence, but little is known about their ageing mechanisms in cotton.

**Results:**

In this study, a group III WRKY TF, *GhWRKY27*, was isolated and characterized. The expression of *GhWRKY27* was induced by leaf senescence and was higher in an early-ageing cotton variety than in a non-early-ageing cotton variety. Overexpression of *GhWRKY27* in *Arabidopsis* promoted leaf senescence, as determined by reduced chlorophyll content and elevated expression of senescence-associated genes (SAGs). Yeast two-hybrid (Y2H) and bimolecular fluorescence complementation (BiFC) assays showed that *GhWRKY27* interacted with an MYB TF, *GhTT2*. Putative target genes of *GhWRKY27* were identified via chromatin immunoprecipitation followed by sequencing (ChIP-seq). Yeast one-hybrid (Y1H) assay and electrophoretic mobility shift assay (EMSA) revealed that *GhWRKY27* binds directly to the promoters of cytochrome P450 94C1 (*GhCYP94C1*) and ripening-related protein 2 (*GhRipen2–2*). In addition, the expression patterns of *GhTT2*, *GhCYP94C1* and *GhRipen2–2* were identified during leaf senescence. Transient dual-luciferase reporter assay indicated that *GhWRKY27* could activate the expression of *GhCYP94C1* and *GhRipen2–2*.

**Conclusions:**

Our work lays the foundation for further study of the functional roles of WRKY genes during leaf senescence in cotton. In addition, our data provide new insights into the senescence-associated mechanisms of WRKY genes in cotton.

**Electronic supplementary material:**

The online version of this article (10.1186/s12870-019-1688-z) contains supplementary material, which is available to authorized users.

## Background

Leaf senescence is the final stage of plant development and is influenced by both internal factors and environmental factors [1, 2]. When these factors are detected by plants, signal transduction occurs, causing changes in gene expression and physiological indices, eventually leading to plant senescence [3]. Although senescence can maximize the plant’s use of its own resources, early and abnormal senescence not only shortens the plant lifespan but also decreases crop yield and quality [4–6]. Therefore, it is essential to study the genes involved in mediating leaf senescence, which will be highly useful for crop genetic breeding.

Analysis of the senescence-associated transcriptome has shown that the expression of many genes can be strongly induced by leaf senescence. Among these genes, WRKY genes constitute the second largest family of transcription factors (TFs), which is smaller only than the family of NAC TFs, strongly demonstrating that WRKY TFs play important roles during leaf senescence [7, 8]. WRKY TFs are named for their conserved WRKY domains and can be classified into three main categories: groups I, II (IIa, IIb, IIc, IId and IIe) and III [9, 10]. These TFs can specifically bind to W-boxes in the promoters of target genes to regulate gene expression and participate in leaf senescence, pathogen defence, stress responses and plant growth and development [11–14]. W-boxes are also found in the promoter regions of many WRKY genes, suggesting the existence of a regulatory network among WRKY TFs and potential functional roles of W-boxes in leaf senescence [13, 15, 16]. Moreover, WRKY TFs can regulate leaf senescence by interacting with a variety of proteins via gibberellic acid (GA)-, jasmonic acid (JA)-, auxin-, and salicylic acid (SA)-mediated signalling pathways [17–19].

Recent functional studies have provided some strong and persuasive evidence that WRKY TFs participate in the senescence-associated regulatory network. Several WRKY genes, such as *AtWRKY6* [20], *AtWRKY18* [21], *AtWRKY22* [22], *AtWRKY45* [17], *AtWRKY53* [23], *AtWRKY54* [24], *AtWRKY57* [18], *AtWRKY70* [25] and *AtWRKY75* [26], have been shown to be SAGs in *Arabidopsis*, and their corresponding regulatory mechanisms have been elucidated at the molecular level. For example, a recent report indicated that a positive regulator of ageing, *AtWRKY75*, is involved in a tripartite amplification loop in which mutual promotion of *AtWRKY75*, SA and reactive oxygen species (ROS) occurs via different mechanisms [26]. Overexpression of *AtWRKY45* promotes leaf senescence, and *AtWRKY45* can interact with the DELLA protein *RGL1* to modulate leaf senescence via a GA-mediated signalling pathway [17]. The mechanisms by which WRKY TFs regulate ageing have also been identified in other species, such as Chinese flowering cabbage, litchi and rice [14, 27, 28]. *BrWRKY65* has been shown to be upregulated during postharvest leaf senescence in the economically important leafy vegetable Chinese flowering cabbage and to directly bind to the downstream SAGs *BrNYC1*, *BrSGR1* and *BrDIN1* [14]. In litchi, *LcNAC1* interacts with *LcWRKY1* to form a complex that antagonistically regulates *LcAOX1a* expression in postharvest litchi fruit senescence [27]. *OsWRKY42* represses the expression of *OsMT1d* by inducing ROS production, thus promoting leaf senescence in rice [28].

Cotton is an important economic crop as well as the dominant raw material of the textile industry. The cultivation of short-season cotton varieties is an effective way to solve the contradiction between grain and cotton. Early maturity is an important trait of short-season cotton varieties. However, some early-maturing cotton varieties are prone to pro-senescence [29]. To overcome this challenge, identification and functional characterization of genes involved in leaf senescence are important for cultivating early-maturing but non-early-ageing cotton varieties. Previously, we conducted a transcriptomic analysis to identify differentially expressed genes during leaf senescence in cotton [30]. It was found that leaf senescence induced a considerable proportion of genes encoding TFs, including WRKY TFs [30]. To date, very few WRKY TFs have been functionally characterized during leaf senescence, and little is known about their molecular mechanisms and the signalling pathways associated with leaf senescence in cotton. In the present study, we performed molecular and genetic assays to identify the functional roles of *GhWRKY27* in age-triggered leaf senescence. Overexpression of *GhWRKY27* in *Arabidopsis* can promote leaf senescence. In addition, *GhTT2* can physically interact with *GhWRKY27*, and *GhWRKY27* targets *GhCYP94C1* and *GhRipen2–2*. *GhWRKY27* activates the transcriptional expression of *GhCYP94C1* and *GhRipen2–2* in the regulatory pathway*.* Therefore, *GhWRKY27* may be a potential ageing-related factor and participate in leaf senescence together with *GhTT2*, *GhCYP94C1* and *GhRipen2–2*. Our studies provide new mechanistic insight into the roles of WRKY genes during leaf senescence in cotton.

## Results

### Isolation and sequence analysis of *GhWRKY27*

**Fig. 1:**
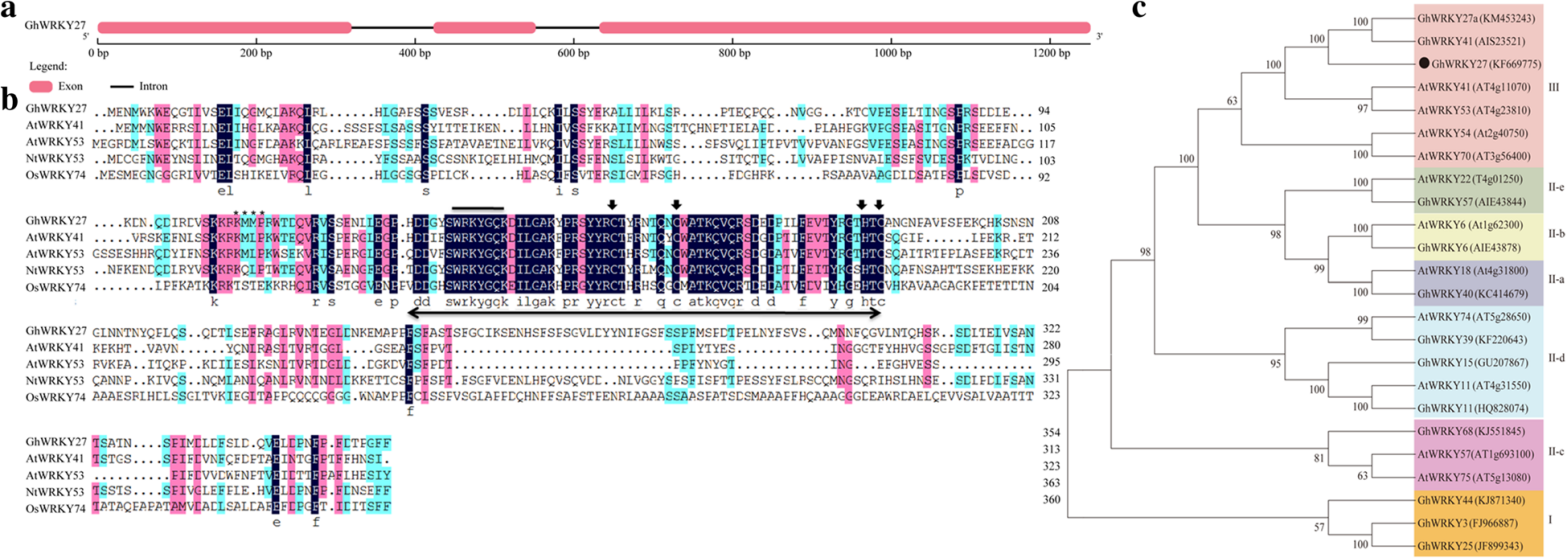
Sequence characteristics and phylogenetic analyses of *GhWRKY27*. **a** Intron-exon structure of *GhWRKY27*. Exons are indicated by thick red lines, and introns are indicated by black lines. **b** Multiple sequence alignment analysis of the *GhWRKY27* protein with its homologues in other species. The WRKY domain, composed of approximately 60 amino acids, is represented by a double-headed arrow. The highly conserved core amino acid sequence, WRKYGQK, in the WRKY domain is indicated with a horizontal line. The C and H residues in the zinc-finger motif are indicated by arrows. **c** Phylogenetic analysis of *GhWRKY27* with other WRKY proteins from cotton and *Arabidopsis*. *GhWRKY27* is indicated by a dot. Gh, *Gossypium hirsutum*; At, *Arabidopsis thaliana*; Nt, *Nicotiana tabacum*; Os, *Oryza sativa*

We previously showed that the *GhWRKY27* (accession KF669775) gene is induced by leaf senescence and highly expressed in senescent leaves based on a transcriptome database [30, 31]. Thus, *GhWRKY27* was selected for further functional characterization. The open reading frame (ORF) and genomic DNA fragments of *GhWRKY27* were isolated from the cotton variety CCRI74. The ORF of *GhWRKY27* is 1068 bp long and encodes 355 amino acid residues. The molecular weight (Mw) of the deduced protein is 40.06 kDa, and the isoelectric point (pI) is 5.48. The *GhWRKY27* gene contains three exons and two introns (Fig. 1a). Multiple sequence alignment of the *GhWRKY27* protein with its related proteins showed that the *GhWRKY27* protein shares 41.30, 36.43, 49.59, and 28.61% identity with *AtWRKY41* (AT4G11070), *AtWRKY53* (AT4G23810), *NtWRKY53* (XP_016508373) and *OsWRKY74* (AAT84161), respectively. In addition, the putative *GhWRKY27* protein contains a typical WRKY domain that includes the highly conserved amino acid sequence “WRKYGQK” and a C-X_7_-C-X_23_-H-X_1_-C zinc-finger motif (Fig. 1b), which is a typical feature of group III members according to Eulgem et al. [9]. A phylogenetic tree was constructed to assess the evolutionary relationships of the *GhWRKY27* protein with other WRKY proteins from cotton and *Arabidopsis*. Phylogenetic analysis showed that *GhWRKY27* clusters with proteins that are reported group III members (Fig. 1c).

### Expression patterns of *GhWRKY27* during leaf senescence

**Fig. 2:**
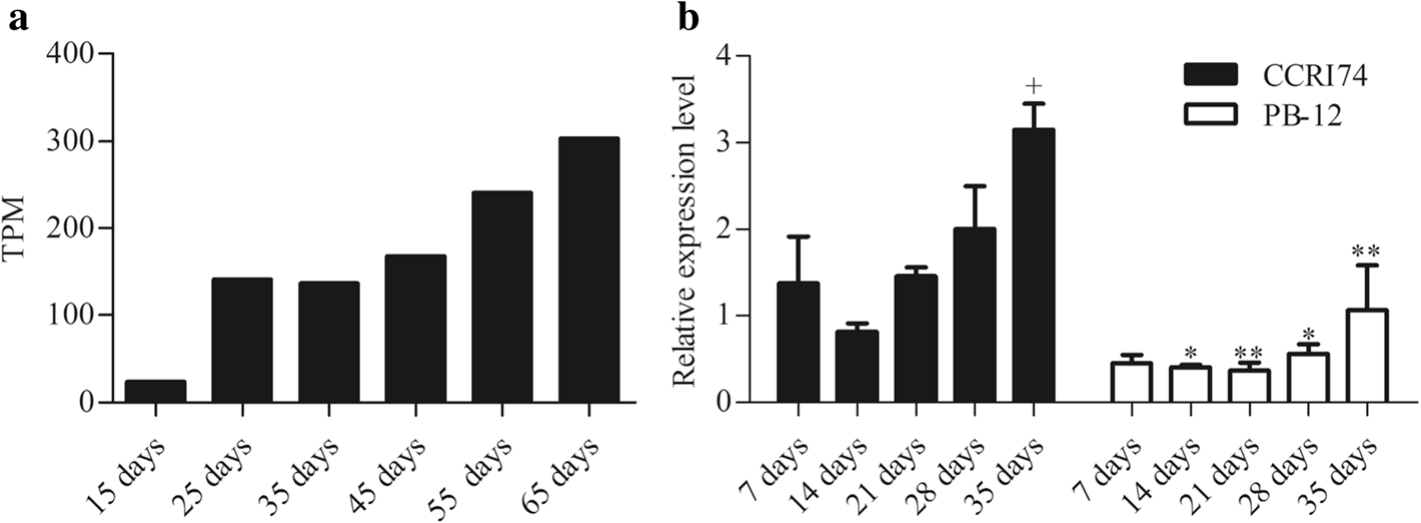
Expression patterns of *GhWRKY27* during leaf senescence in cotton. **a** Expression profiles of the *GhWRKY27* gene in 15-, 25-, 35-, 45-, 55-, and 65-day-old leaves of the CCRI36 cotton variety during leaf senescence using RNA-seq data [30]. The expression values were normalized using the transcript per million clean tags (TPM) algorithm. **b** Transcript levels of *GhWRKY27* in the early-ageing cotton variety CCRI74 and non-early-ageing cotton variety PB-12. Values represent the means ± standard deviations (SDs) from three replicates. The significance of the data was determined using Student’s t-test. “+” represents a comparison with 7 days in the same variety (^+^*P* < 0.05). “**” and “*” represent a comparison between the same days of different varieties (^**^P < 0.01 and ^*^P < 0.05)

To understand the potential role of *GhWRKY27* during leaf senescence, the transcript levels of *GhWRKY27* were determined at different stages of leaf senescence. Using the expression profile data published by Lin et al. [30], the expression levels of *GhWRKY27* were found to be gradually upregulated during leaf senescence in 15-, 25-, 35-, 45-, 55-, and 65-day-old leaves of the CCRI36 variety (Fig. 2a). The transcript accumulation of *GhWRKY27* was further detected in the early-ageing cotton variety CCRI74 and the non-early-ageing cotton variety PB-12 at different stages of leaf senescence. The expression level of *GhWRKY27* gradually increased in both varieties and was significantly lower in PB-12 than in CCRI74 in 14- to 35-day-old leaves (Fig. 2b). In addition, the transcript level of *GhWRKY27* was significantly higher in 35-day-old leaves than in 7-day-old leaves in CCRI74. However, there was no difference in expression at different stages of leaf senescence in PB-12 (Fig. 2b).

### Overexpression of *GhWRKY27* promotes leaf senescence in transgenic *Arabidopsis* plants

**Fig. 3:**
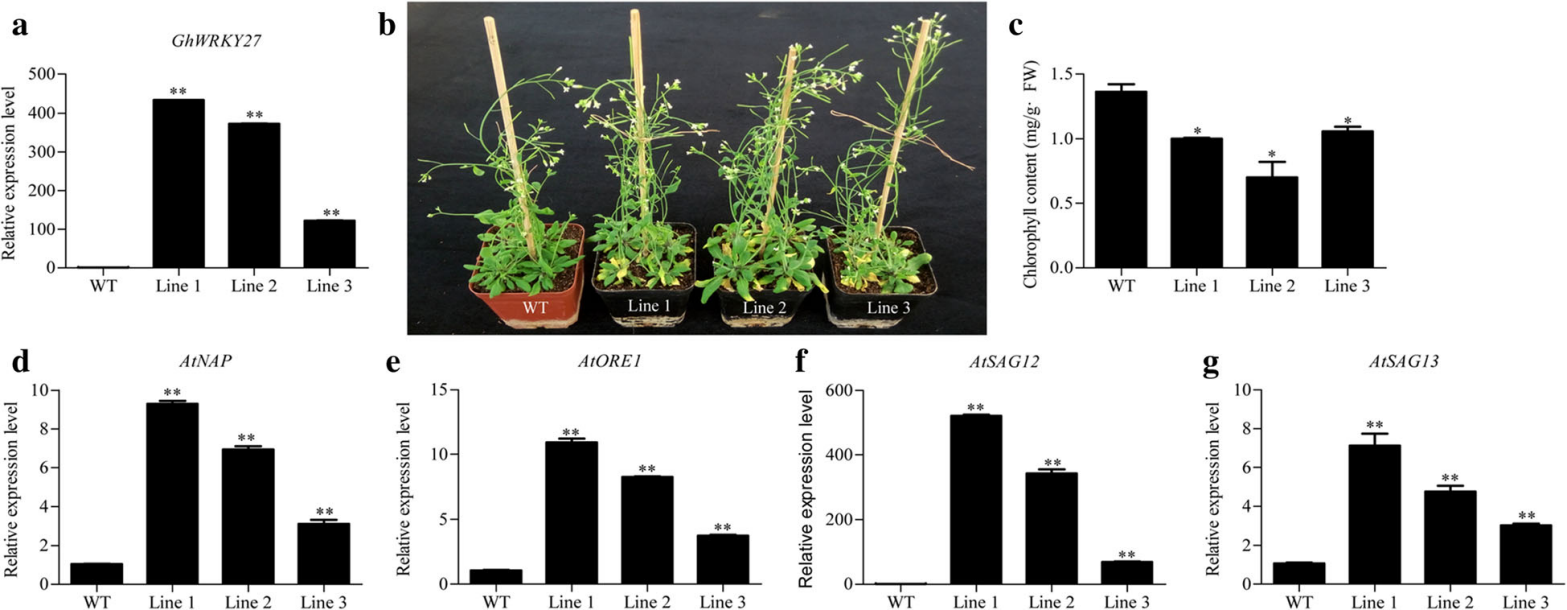
The *GhWRKY27* gene accelerates age-triggered leaf senescence in transgenic *Arabidopsis*. **a** qRT-PCR analysis of *GhWRKY27* transcript levels in WT and three transgenic lines. **b** Senescence phenotypes of WT plants and three transgenic lines. **c** Chlorophyll content of WT plants and three transgenic lines. **d-g** qRT-PCR analysis of the transcript levels of the SAGs *AtNAP*, *AtORE1*, *AtSAG12* and *AtSAG13* in *Arabidopsis*. *AtUBQ10* was employed as a reference gene. Values represent the means ± SDs. The significance of the data was determined using Student’s t-test (^**^P < 0.01). The experiment was conducted with three repetitions

To further investigate the functional role of *GhWRKY27* in leaf senescence, *GhWRKY27* was transformed into *Arabidopsis*, and three independent transgenic lines were confirmed by quantitative real-time PCR (qRT-PCR) (Fig. 3a). The wild-type (WT) and transgenic plants were cultured on 1/2 MS medium. Two weeks later, the plants were transplanted to nutrient-rich soil in a greenhouse to observe the natural senescence phenotype. The transgenic lines exhibited a severe leaf senescence phenotype compared with that of WT plants (Fig. 3b). The chlorophyll content was significantly lower in transgenic plants than in WT plants (Fig. 3c). The transcript levels of four SAGs associated with positive regulation of leaf senescence (*AtNAP*, *AtORE1*, *AtSAG12* and *AtSAG13*) were found to be significantly higher in the transgenic plants than in WT plants (Fig. 3d-g). In addition, the expression trends of these genes were consistent with those of *GhWRKY27* in the three transgenic lines (Fig. 3d-g).

### *GhTT2* is an interacting partner of *GhWRKY27* in yeast cells and *Arabidopsis* protoplasts

We constructed a yeast two-hybrid (Y2H) cDNA library using senescent cotton leaves and performed library screening using *GhWRKY27* as bait to screen its interacting partners. Through library screening, approximately 164 positive colonies were isolated and sequenced. After discarding repeat sequences, 67 potential interacting proteins were identified. Functional annotation analysis showed that these candidate proteins were mainly involved in signal transduction, stress responses and regulation of growth and development (Additional file 1: Table S1).

**Fig. 4:**
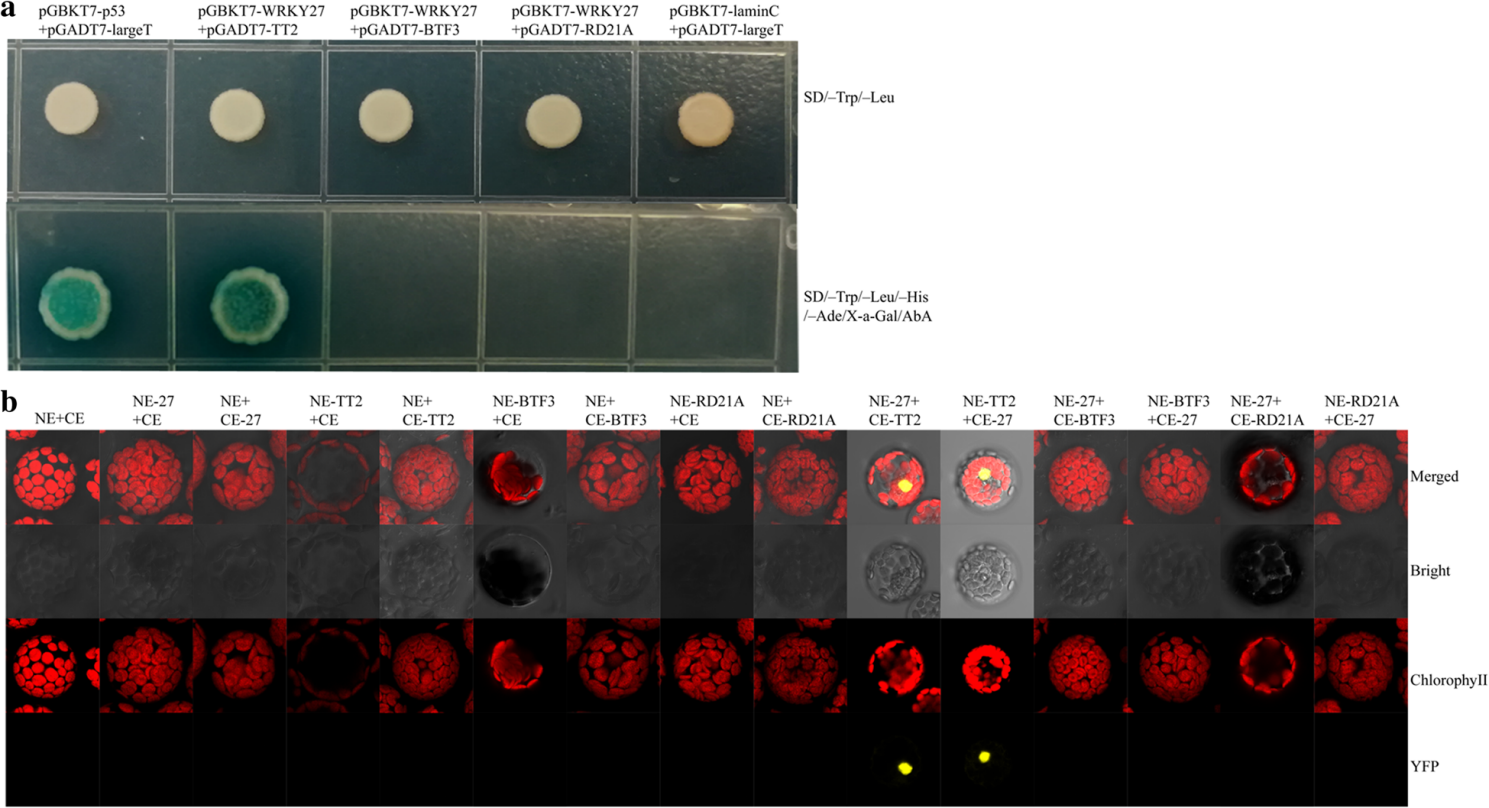
The interaction of *GhWRKY27* with *GhTT2*, *GhBTF3* and *GhRD21A* was determined in yeast cells and *Arabidopsis* mesophyll protoplasts. **a** Physical interaction of *GhTT2*, *GhBTF3* and *GhRD21A* with *GhWRKY27* in yeast cells. pGBKT7-p53 + pGADT7-largeT and pGBKT7-laminC + pGADT7-largeT were used as positive and negative controls, respectively. **b** The interaction of *GhTT2*, *GhBTF3* and *GhRD21A* with *GhWRKY27* in *Arabidopsis* mesophyll protoplasts. The yellow signal indicates YFP fluorescence, and the red signal indicates the autofluorescence of chlorophyll. The merged image is a combination of images showing the YFP and chlorophyll signals. No YFP signals were detected in the negative controls

To confirm which proteins interact with *GhWRKY27*, three candidate genes, MYB domain protein gene TT2 (Gh_A07G0140, *GhTT2*), TF BTF3 (Gh_A08G2018, *GhBTF3*) and cysteine proteinase RD21a (Gh_D01G1044, *GhRD21A*), were selected and investigated using a Y2H system. The ORFs of the three genes were cloned into the pGADT7 vector. Each fusion construct and pGBKT7-*GhWRKY27* plasmid was co-transformed into the yeast strain Y2HGold. Our results showed that the co-transformed yeast cells containing the positive control pGBKT7-p53 + pGADT7-largeT and the experimental group pGBKT7-*GhWRKY27 +* pGADT7-*GhTT2* could grow well on SD/−Trp/−Leu/-His medium and turned blue on SD/−Trp/−Leu/−His/−Ade/X-a-Gal/AbA medium. However, no interaction was found in pGBKT7-*GhWRKY27 +* pGADT7-*GhBTF3*, pGBKT7-*GhWRKY27 +* pGADT7-*GhRD21A* and the negative control pGBKT7-laminC + pGADT7-largeT in yeast cells (Fig. 4a). Furthermore, the remaining genes in Additional file 1: Table S1 could not interact with *GhWRKY27* (data not shown).

To further confirm the interactions of *GhWRKY27* with the three candidate proteins in plant cells, bimolecular fluorescence complementation (BiFC) assays were conducted in *Arabidopsis* protoplasts. The results showed that *GhTT2* interacted with *GhWRKY27* by emitting a yellow fluorescent signal in the nucleus, while *GhBTF3* and *GhRD21A* did not exhibit any fluorescence (Fig. 4b). In addition, no yellow fluorescent signal was detectable when two empty vectors and one empty vector with a fusion construct were applied (Fig. 4b).

### Screening for genomic binding sites of *GhWRKY27* using a chromatin immunoprecipitation followed by sequencing (ChIP-seq) assay

**Fig. 5:**
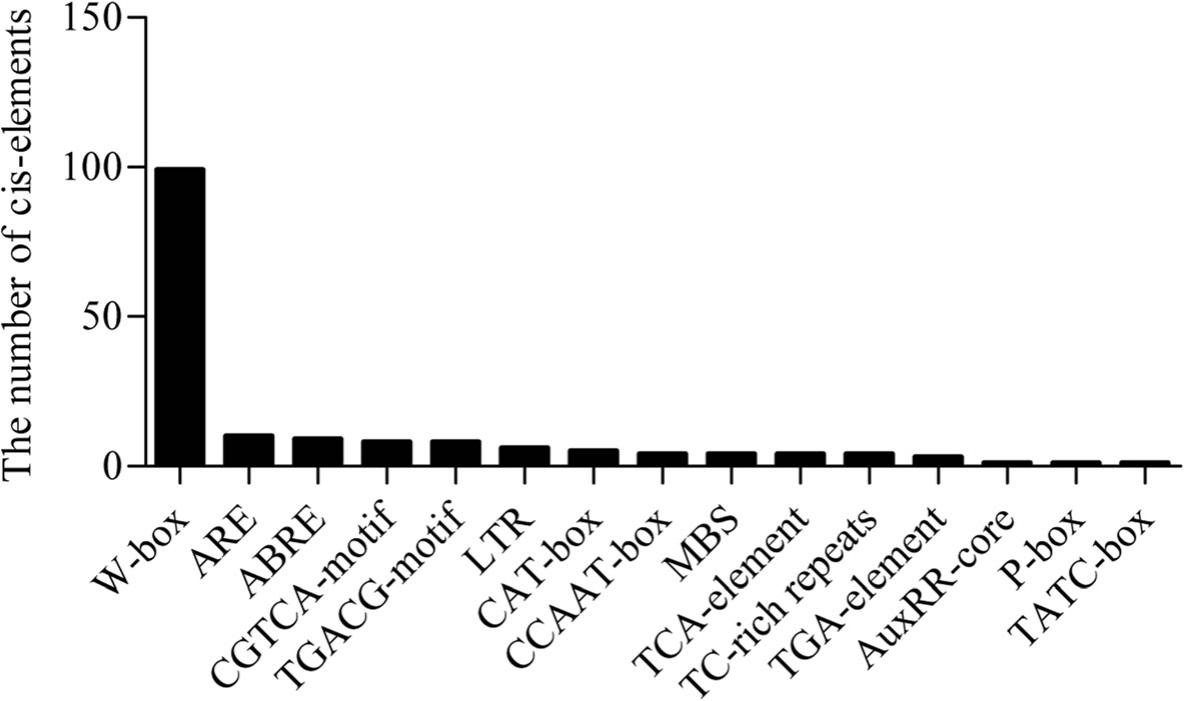
The cis-element accumulation in the ChIP-seq results

ChIP-seq was used to identify the locations in the genome to which the *GhWRKY27* protein can bind. The locations were characterized by significant numbers of mapped reads (peaks) [32]. The ChIP-seq results showed that the peaks came from 98 different target promoter regions, and the fold enrichment of the peaks varied from 15.59 (Gh_A10G0788) to 127.86 (Gh_A07G1258) (Additional file 2: Table S2). In addition, W-boxes were abundant in the sequencing data and were the most numerous among the cis-elements (Fig. 5). In the 1500-bp promoter region, 94 of the genes contained W-boxes, and several genes contained one or more W-boxes (Additional file 2: Table S2). The putative direct target genes included TFs, stress- and defence-related genes, growth- and development-related genes, photosynthesis genes, enzyme-encoding genes, ion transport-related genes and several unknown genes (Additional file 2: Table S2).

### Yeast one-hybrid (Y1H) assay and electrophoretic mobility shift assay (EMSA) show that *GhWRKY27* binds to the promoters of *GhCYP94C1* and *GhRipen2–2*

**Fig. 6:**
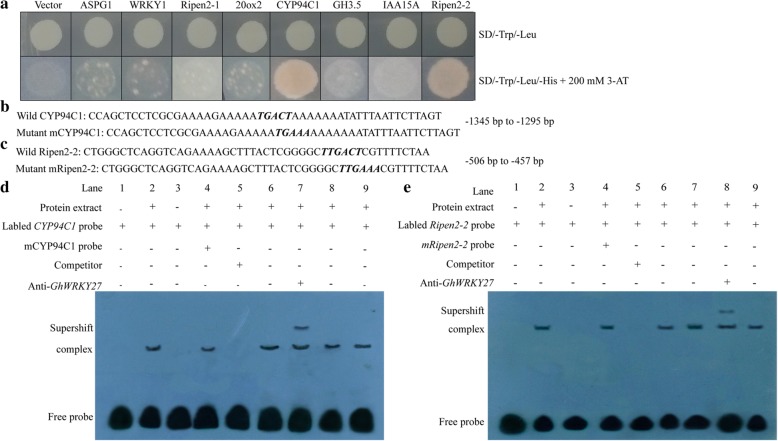
*GhWRKY27* directly targets the promoters of *GhCYP94C1* and *GhRipen2–2*. **a** Interaction between *GhWRKY27* and the candidate target genes *GhASPG1*, *GhWRKY1*, *GhRipen2–1*, *Gh20ox2*, *GhCYP94C1, GhGH3.5*, *GhIAA15A*, and *GhRipen2–2* in Y187 yeast cells. The binding ability of *GhWRKY27* with the target promoter sequences was identified based on the growth status of transformed Y187 yeast cells on SD-TL and SD-TLH + 200 mM 3-AT medium. The combination of pGADT7-*GhWRKY27* and pHIS2 was used as a negative control. **b** Wild and mutated promoter probe sequences of *GhCYP94C1* were used in the EMSA assay. The W-box sequence 5′-TGACT-3′ was mutated to 5′-TGAAA-3′. **c** Wild and mutated promoter probe sequences of *GhRipen2–2* were used in the EMSA assay. The W-box sequence 5′-TTGACT-3′ was mutated to 5′-TTGAAA-3′. **d** *GhWRKY27* binds to the W-boxes in the promoter of *GhCYP94C1* in the EMSA assay. **e** *GhWRKY27* binds to the W-boxes in the promoter of *GhRipen2–2* in the EMSA assay. A monoclonal antibody against *GhWRKY27* (anti-*GhWRKY27*) was employed in the supershift assay. “–” indicates absence, while “+” indicates presence

To identify the downstream target genes participating in the regulatory network, 8 putative target genes were selected and validated by using the Y1H system. These genes encode hormone-related enzymes (*GhIAA15A*, *Gh20ox2* and *GhGH3.5*), TFs (*GhWRKY1*), ripening-related proteins (*GhRipen2–1* and *GhRipen2–2*), aspartic protease (*GhASPG1*) and cytochrome P450 (*GhCYP94C1*). Three tandem copies from the peak sequences, which represent the most likely binding sites in the promoter regions, were fused to the pHIS2 bait vector (Additional file 3: Table S3). *GhWRKY27* was inserted into the pGADT7 prey vector. The prey construct was co-transformed into Y187 yeast cells with the bait carriers. The results showed that the transformation products harbouring the combination of *GhWRKY27* with the specific promoter regions of *GhCYP94C1* and *GhRipen2–2* grew well on SD/−Trp/−Leu + 200 mM 3-AT selective medium, whereas the negative control and other candidate genes did not (Fig. 6a).

The direct binding of the *GhWRKY27* protein to the *GhCYP94C1* and *GhRipen2–2* promoters was further confirmed via EMSA. As shown in Fig. 6b and c, the wild and mutant probe sequences, which were the WRKY recognition regions containing the W-box, were present within the − 1345 bp to − 1295 bp upstream region of the ATG initiation codon of *GhCYP94C1* and the − 506 bp to − 457 bp of *GhRipen2–2*. The EMSA results revealed that the *GhWRKY27* protein was able to recognize and bind the labelled wild probes of *GhCYP94C1* to cause a mobility shift (Fig. 6d; lanes 2, 6, 8, 9). In addition, in the presence of the labelled wild probe of *GhCYP94C1*, the unlabelled mutant probe did not affect the generation of the lagging band when overdosed at the same time (Fig. 6d; lane 4). However, the band was effectively abolished when the unlabelled wild probe of *GhCYP94C1* was overdosed as a cold competitor (Fig. 6d; lane 5). In this case, most labelled wild probes were replaced by unlabelled wild probes. Therefore, the *GhWRKY27* protein mainly bound to the competitors (unlabelled wild probes), leading to the disappearance of the band (Fig. 6d; lane 5). Furthermore, anti-*GhWRKY27* was employed for supershift detection. When anti-*GhWRKY27* was added, the antibody-protein-DNA complex formed a retarded band (Fig. 6d; lane 7). Similar results were found in *GhRipen2–2* (Fig. 6e)*.* Our findings clearly showed that the *GhWRKY27* protein bound specifically to the W-box in the promoter regions of *GhCYP94C1* and *GhRipen2–2*.

### The expression patterns of *GhTT2, GhCYP94C1* and *GhRipen2–2* at different stages of leaf senescence

**Fig. 7:**
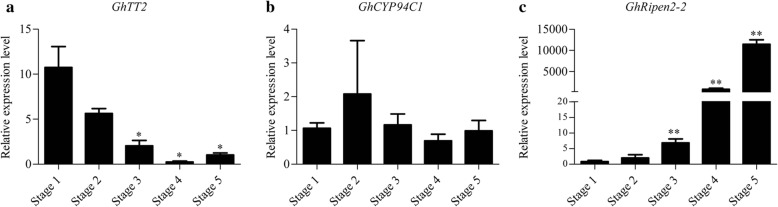
Expression patterns of *GhTT2*, *GhCYP94C1* and *GhRipen2–2* at different stages of leaf senescence. **a** Transcript levels of *GhTT2* during leaf senescence. **b** Transcript levels of *GhCYP94C1* during leaf senescence. **c** Transcript levels of *GhRipen2–2* during leaf senescence. Stage 1 to stage 5 indicate different stages of cotton leaves with different senescence areas, as described previously [35]. *GhActin* was used as an internal reference. Values represent the means ± SDs. The significance of the data was determined using Student’s t-test (^**^P < 0.01 and ^*^P < 0.05). The experiment was conducted with three replications

It has been demonstrated that the genes *GhTT2*, *GhCYP94C1* and *GhRipen2–2* interact with *GhWRKY27*. *GhWRKY27* is a positive regulator of leaf senescence, which motivated us to study the roles of *GhTT2*, *GhCYP94C1* and *GhRipen2–2* during leaf senescence. The expression levels of *GhTT2*, *GhCYP94C1* and *GhRipen2–2* were examined at different stages of leaf senescence. The results showed that the transcript abundance of *GhTT2* decreased gradually as the leaf aged and that the expression levels of *GhTT2* were significantly lower in Stages 3–5 than in Stage 1 (Fig. 7a). The transcript levels of *GhCYP94C1* showed no obvious differences among the different stages (Fig. 7b). However, the expression levels of the *GhRipen2–2* gene gradually increased with the senescence of the leaves and were 10,000 times higher at Stage 5 than at Stage 1; this difference was highly significant (Fig. 7c).

### *GhWRKY27* increased transcriptional expression of *GhCYP94C1* and *GhRipen2–2*

**Fig. 8:**
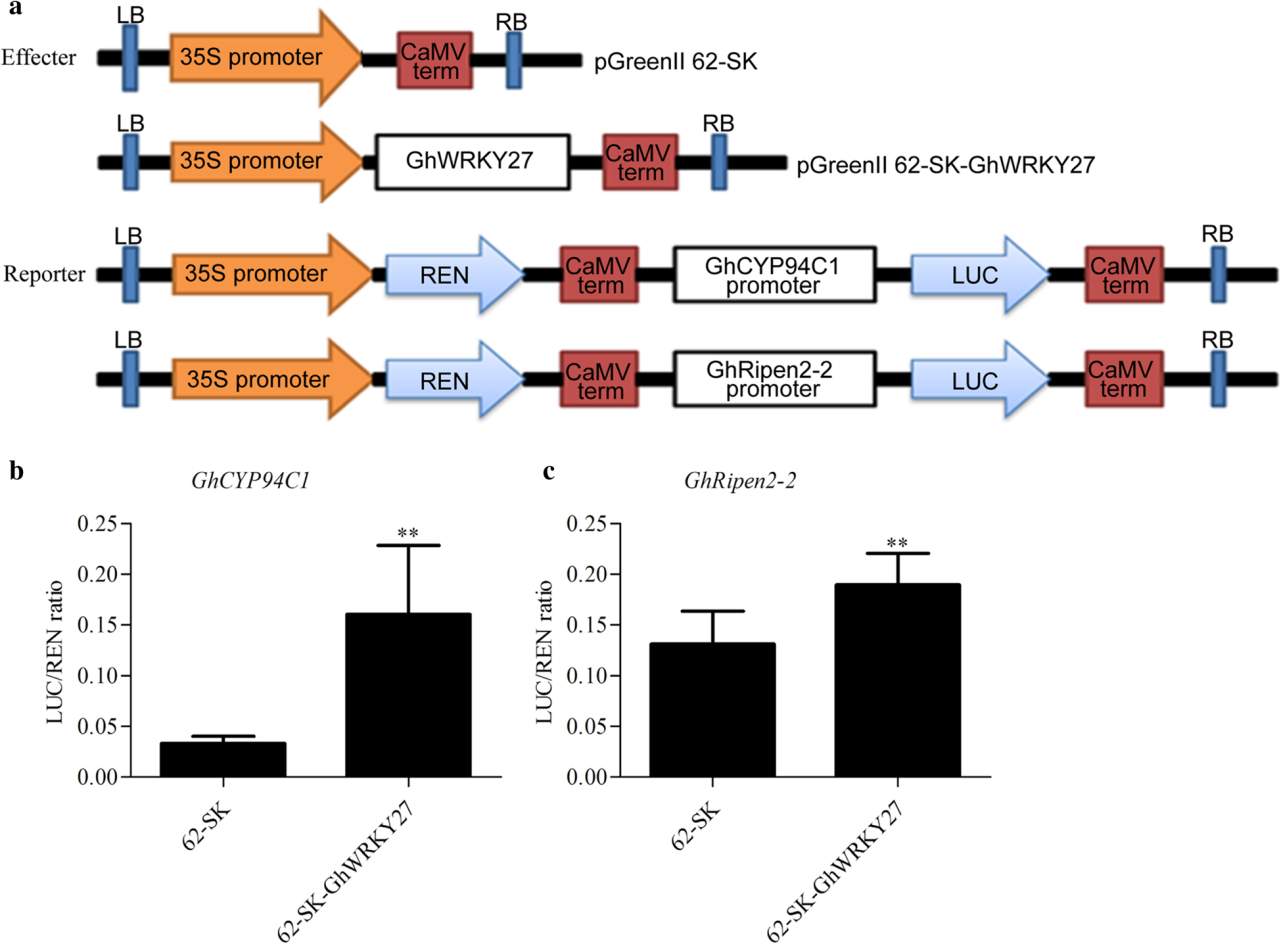
*GhWRKY27* activated the expression of *GhCYP94C1* and *GhRipen2–2* in the dual-luciferase reporter assay. **a** Sketch of the effecter and reporter constructs. The *GhWRKY27* was cloned into the effecter vector pGreenII 62-SK. The promoters of *GhCYP94C1* and *GhRipen2–2* were cloned into the reporter vector pGreenII 0800-LUC. **b** *GhWRKY27* activated the expression of *GhCYP94C1*. **c** *GhWRKY27* activated the expression of *GhRipen2–2*. The data were indicated by the ratio of LUC to REN. The values represent the means ± SDs of six independent repeats. The significance of the data was determined using Student’s t-test (^**^P < 0.01)

A transient dual-luciferase assay was performed in tobacco leaves to elucidate the functional role of *GhWRKY27* in regulating the expression of *GhCYP94C1* and *GhRipen2–2* in vivo. The experiment was conducted using a double reporter plasmid, pGreenII0800-LUC, containing the REN luciferase driven by the 35S promoter and the LUC luciferase driven by the *GhCYP94C1* or *GhRipen2–2* promoter. In addition, the assay includes an effecter plasmid, pGreenII62-SK, expressing the *GhWRKY27* TF. The constructs are shown in Fig. 8a. The ratio of LUC to REN was used to reflect the transcriptional activity. The results showed that compared with the control, *GhWRKY27* activated the *GhCYP94C1* and *GhRipen2–2* promoters by significantly increasing the LUC/REN ratio (Fig. 8b, c), suggesting that *GhWRKY27* activated the expression of *GhCYP94C1* and *GhRipen2–2*.

## Discussion

Many important agronomic traits, such as crop yield and quality, are affected by leaf senescence. However, the regulatory mechanism of leaf senescence in cotton remains poorly understood. In our previous work, RNA-seq was performed using leaves in different stages of senescence to comprehensively analyse gene expression in cotton [30]. Similar to the transcriptomic analysis of leaf senescence in *Arabidopsis*, our transcriptomic analysis revealed that the WRKY gene family is one of the largest TF families induced by leaf senescence [7, 30]. Subsequently, a genome-wide analysis of the WRKY gene family was performed in cotton, and the specific functional roles of the WRKY gene family during leaf senescence were explored [31]. Previous reports have shown that members of the group III WRKY subfamily participate in leaf senescence [31]. In *Arabidopsis*, several ageing-related WRKY genes have been studied, among which *AtWRKY30*, *AtWRKY53*, *AtWRKY54* and *AtWRKY70* belong to the group III subfamily [24]. Given the important roles of the group III subfamily in leaf senescence, the group III subfamily was further analysed in cotton, and *GhWRKY27* was identified as a member of this subfamily [33]. In the present study, multiple sequence alignment and evolutionary analysis further demonstrated that *GhWRKY27* belongs to the group III subfamily.

Leaf senescence is basically regulated by the developmental age and influenced by various internal and external factors, causing degradation of macromolecules and translocation of nutrients, eventually leading to cell death [2]. In the present study, *GhWRKY27* was closely related to group III members. Previous reports showed that group III genes *AtWRKY30/53/54/70* are senescence regulators in *Arabidopsis* [24]. For example, *AtWRK70* negatively regulates developmental senescence and has been found to be involved in SA- and JA/ETH-mediated signalling pathways based on examining transgenic and mutant plants [25]. In addition, a positive regulator of leaf senescence, *AtWRKY53*, which shows the highest similarity with *GhWRKY27*, functions as a convergence node between senescence and H_2_O_2_-, SA- and JA-related stress response [23]. We found that the transcript levels of *GhWRKY27* increased as leaf senescence progressed. Moreover, in the early-ageing cotton variety, the expression level of *GhWRKY27* was significantly higher than that in the non-early-ageing cotton variety. Guo et al. showed that the expression of *AtWRKY75* increases gradually with leaf ageing and that overexpression of *AtWRKY75* in *Arabidopsis* plants accelerates leaf senescence [26]. In addition, *GhNAC12* [34] and *GhWRKY17* [35] have been found to be highly expressed in an early-ageing cotton variety, and leaf senescence is triggered in *Arabidopsis* plants transgenic for these genes. The high expression level of the *GhWRKY27* gene in senescent leaves and in the early-ageing variety indicates the potential role of *GhWRKY27* in leaf senescence. SAGs have been demonstrated to serve as indicators of leaf senescence [2, 17, 36]. SAGs such as *AtNAP* have been found to be upregulated during leaf senescence and to positively regulate leaf senescence in transgenic *Arabidopsis* [37]. *AtORE1*, an NAC TF in *Arabidopsis*, is involved in regulatory networks of age-dependent and salt-promoted senescence [38]. *AtSAG12* and *AtSAG13*, which encode cysteine proteases, are upregulated during developmental senescence [17]. Four marker genes of leaf senescence, *AtNAP*, *AtORE1*, *AtSAG12* and *AtSAG13*, are significantly upregulated in transgenic *Arabidopsis*, and overexpression of *GhWRKY27* accelerates leaf senescence in transgenic *Arabidopsis* plants, which further demonstrates the positive role of *GhWRKY27* during leaf senescence. In our previous work, the expression level of *GhWRKY27* was significantly elevated after SA treatment [33], suggesting the important role of this gene in SA-related stress response.

Some WRKYs regulate leaf senescence by interacting with other proteins [24]. For example, JAZ4/8 and IAA29, which are repressors of the JA and indole-3-acetic acid (IAA) signalling pathways, respectively, competitively interact with *AtWRKY57* to negatively regulate JA-induced leaf senescence [18]. The JA-inducible protein EPITHIOSPECIFYING SENESCENCE REGULATOR (ESR/ESP) and the HECT-domain E3 ubiquitin ligase (UPL5) have been determined to interact with the positive ageing regulator *AtWRKY53* via distinct mechanisms [19, 39]. Here, we screened for putative interaction partners of *GhWRKY27* using a Y2H system. *GhTT2*, an MYB family protein, was found to interact with *GhWRKY27*. Previous studies have demonstrated that *TT2*, which encodes an R2R3 MYB domain protein, is mainly involved in the regulation of proanthocyanidin metabolism [40, 41]. In *Arabidopsis*, *AtTT2/AtMYB123* positively regulates anthocyanin metabolism and is also involved in flavonoid-related pathways [42]. In cotton, two TT2-type MYB genes, *GhTT2-3A* and *GhTT2_A07*, are associated with brown cotton fibres by influencing the accumulation of proanthocyanidin [43, 44]. However, MYB TFs such as *AtMYBL* [45] and *AtMYB44* [46] have been shown to be involved in leaf senescence, and *AtMYBL* overexpression in *Arabidopsis* plants promotes leaf senescence [45]. Usually, CAB (chlorophyll a/b-binding protein) is used as a senescent marker gene, and the expression level of CAB gradually decreases with leaf senescence [47]. In our work, similar to the CAB gene, *GhTT2* showed decreased expression during leaf senescence, indicating that *GhTT2* might be involved in the early stage of leaf senescence. However, the relationship between *GhTT2* and leaf senescence should be further clarified in transgenic materials.

W-box cis-elements in promoter regions have been reported to act as binding motifs for WRKY TFs [48]. The ChIP assay results showed that W-box elements accumulated in the sequencing data and that 94 of the 98 genes contained different numbers of W-boxes. The appearance of W-boxes in the promoter fragments indicated their potential functions as candidate target genes [13]. Some genes possessed one or more W-boxes in their promoter regions. Although a single W-box element in the promoter region is enough for participation in gene expression regulated by the WRKY gene, multiple W-box elements are frequently found to be clustered in promoter regions [9]. We discovered that the promoter regions of several genes contain no W-box elements. Previous studies have shown that *AtWRKY6* and *AtWRKY53* can directly target the promoter region of *AtWRKY42*, which lacks W-boxes [12, 13]. These findings indicate that the targets of WRKY TFs may be indirectly regulated or may be regulated via different elements in promoter regions [12, 13]. Therefore, it remains difficult to determine whether the *GhWRKY27* gene directly targets candidate genes based solely on the presence or absence of W-boxes. The peak sequences of the potential target genes (*GhASPG1*, *GhWRKY1*, *GhRipen2–1*, *Gh20ox2*, *GhCYP94C1*, *GhGH3.5*, *GhIAA15A* and *GhRipen2–2*) were used as candidate sequences for Y1H verification. Whether the *GhWRKY27* gene can bind to W-boxes in other regions of their promoters remains to be determined.

Our results showed that *GhCYP94C1* and *GhRipen2–2* are targets of *GhWRKY27*. *GhCYP94C1* is a member of the cytochrome P450 family. Some studies have shown that cytochrome P450 genes such as *CYP89A9* [49], *CYP74C9* [50], *CYP82E4* [51] and *CYP76C2* [52] are involved in plant leaf senescence. For example, *CYP89A9*, a cytochrome P450 monooxygenase, plays an important role in the accumulation of nonfluorescent dioxobilin-type chlorophyll catabolites (NDCC), a major chlorophyll degradation product, during leaf senescence in *Arabidopsis* [49]. In our study, *GhWRKY27* activated the expression of *GhCPY91C1* in the dual-luciferase reporter system. However, we observed no differences in *GhCYP94C1* expression at different stages of leaf senescence and the Ct values were greater than 33 at every stage. Tissue-specific expression analysis showed that *GhCYP94C1* has very high expression levels in flower organs and very low levels in leaves (Additional file 4: Figure S1). The expression of *GhCYP94C1* was further examined at different stages of flower bud development in different maturing varieties. The expression level of *GhCYP94C1* is higher in early maturing varieties than in non-early maturing varieties and has the highest expression levels at the 3-leaf stage (flower bud differentiation stage) in Yanzao and Guoxin11 (Additional file 5: Figure S2). These results suggested that *GhCYP94C1* might be associated with cotton maturity and flower bud differentiation. Early maturity of cotton is often accompanied by pro-senescence [29]. We speculate that *GhCYP94C1* might affect cotton maturity via affecting reproductive growth, thereby affecting the senescence of cotton. However, the precise functions of *GhCYP94C1* should be clarified by further studies.

However, no studies have yet addressed *GhRipen2–2*-related genes in cotton or their homologues in *Arabidopsis*, indicating that *GhRipen2–2* might be a novel gene that requires further exploration in cotton. In addition, the expression of *GhRipen2–2* was significantly induced during leaf senescence and activated by *GhWRKY27*, suggesting its potential roles in leaf senescence and ageing regulatory pathways.

## Conclusions

In summary, a group III WRKY TF, *GhWRKY27*, was isolated and characterized. The gene expression profiles of cotton leaves from different senescent stages revealed that *GhWRKY27* was induced by leaf senescence and predominantly expressed in the early-ageing cotton variety. Overexpression of *GhWRKY27* accelerated leaf senescence in *Arabidopsis*, accompanied by the elevated accumulation of SAG transcripts. *GhWRKY27* was shown to physically interact with *GhTT2* and bind to the promoters of *GhCYP94C1* and *GhRipen2–2*. *GhTT2*, *GhCYP94C1* and *GhRipen2–2* were differentially expressed at different stages of leaf senescence. In addition, *GhWRKY27* activated the transcriptional expression of *GhCYP94C1* and *GhRipen2–2* during leaf senescence. Our results suggested that *GhWRKY27* may positively regulate leaf senescence via interaction with or transcriptional regulation of other genes. These findings provide novel insight into the regulatory pathways of leaf senescence in cotton and a theoretical basis for genetic improvement of short-season cotton varieties to early-maturing but non-early-ageing cotton varieties.

## Methods

### Plant materials and growth conditions

One early-ageing cotton variety, CCRI74, and two non-early-ageing varieties, PB-12 and Liao4086, were used for leaf senescence analysis. In addition, two early-maturing varieties, CCRI50 and Yanzao, and two non-early-maturing varieties, Guoxin11 and Lu28, were used for maturing analysis. The cotton varieties were planted in the field of the Cotton Research Institute of the Chinese Academy of Agricultural Sciences (Anyang, Henan, China). Different tissues, including root, stems, leaves, petals, pistils, stamens, fibre and ovules were harvested from CCRI74.

To detect gene expression during leaf senescence in cotton, the fourth leaves from the top of the CCRI74 and PB-12 plants were marked at the full-bloom stage. After one week, the leaves were picked weekly at five successive developmental stages (7, 14, 21, 28, and 35 days). In addition, five areas of CCRI74 true leaves with yellowing were harvested according to a previous method [35].

To examine gene expression at different stages of bud differentiation from different maturity varieties, the buds were collected from CCRI50, Yanzao, Guoxin11 and Lu28 at the cotyledon and 1-, 2-, 3-, 4- and 5-leaf stages. Every sample included three repetitions. All samples were quickly frozen in liquid nitrogen and stored at − 80 °C for subsequent experiments.

### Gene clone and sequence analysis

The ORF and genomic DNA sequences of *GhWRKY27* were cloned from the cDNA and DNA of CCRI74 leaves, respectively. The PCR fragments were then inserted into the pMD18-T vector (TAKARA) for sequencing. The intron-exon structure of the *GhWRKY27* gene was generated using the online software GSDS2.0 (http://gsds.cbi.pku.edu.cn/). The conserved domains within the *GhWRKY27* protein were analysed using the online software CDD (https://www.ncbi.nlm.nih.gov/Structure/cdd/wrpsb.cgi). The Mw and pI were predicted with ExPASy (http://web.expasy.org/compute_pi/). Multiple sequence alignment of the *GhWRKY27* protein with its homologues from other species was performed using DNAMAN 6.0 software. A phylogenetic tree was constructed with the MEGA 7.0 program via the neighbour-joining method, with 1000 bootstrap replicates.

### qRT-PCR

Total RNA was isolated using the RNAprep Pure Plant Kit (For Plants) (DP441; Tiangen, Beijing, China). Reverse transcription was conducted using the PrimeScript™ RT Reagent Kit with gDNA Eraser (Perfect Real Time) (RR047A; TaKaRa, Dalian, China). Transcript levels were measured via qRT-PCR with SYBR® Premix Ex Taq™ (Tli RNaseH Plus) (RR420A; TaKaRa) in an ABI 7500 real-time PCR system (Applied Biosystems, Foster City, CA, USA). The *G. hirsutum Actin* (*GhActin*) and *A. thaliana UBQ10* (*AtUBQ10*) genes were used as internal controls. The primers designed with Oligo 7 software are shown in Additional file 6: Table S4. Three biological replicates were performed. The qRT-PCR assays were conducted with three technical replicates.

### Plasmid construction and plant transformation

The ORF of *GhWRKY27* was amplified from the T vector containing *GhWRKY27* and cloned into the pBI121 vector, driven by the *cauliflower mosaic virus* 35S promoter. The recombinant pBI121-*GhWRKY27* plasmid was transformed into the *Agrobacterium tumefaciens* strain LBA4404. The LBA4404 strain harbouring the pBI121-*GhWRKY27* plasmid was transformed into WT *Arabidopsis thaliana* (Colombia-0) using the floral dip method [53]. The transgenic seedlings were screened on 1/2 MS agar medium plates containing kanamycin (50 mg L^− 1^) in a chamber at 22 °C with a 16 h light/8 h dark cycle. The transgenic plants were further confirmed through PCR until the T3 generation. The ageing phenotype was observed in the T3 generation. The rosette leaves were collected for the expression analysis.

### Determination of chlorophyll content

Approximately 0.1 g of rosette leaf sample was placed in a 15 ml tube to which 15 ml of extract (acetone: absolute ethanol = 1:1) was added; the tube was left for 24 h at room temperature in the dark. When the sample was completely white, the absorbance was measured at 645 nm and 663 nm. Chlorophyll content (mg/g· fresh weight) = (20.31D_645_ + 8.03D_663_)(mg L^− 1^)·V(mL)/(fresh weight (g)·1000).

### Y2H library assay

Old CCRI74 leaves were used to construct a cDNA library to screen interacting proteins. The ORF of *GhWRKY27* was amplified and inserted into the pGBKT7 vector containing the GAL4 DNA-binding domain. A Y2H library screening assay was performed following the manufacturer’s protocols (Clontech). The fusion construct pGBKT7-*GhWRKY27* and the cDNA library plasmids were co-transformed into yeast strain Y2HGold competent cells (Clontech). The transformed cells were initially screened on lower-stringency SD/−Trp/−Leu/−His/−Ade agar plates with incubation at 30 °C for 3–5 days. Yeast colonies with diameters greater than 2 mm were patched onto the higher-stringency SD/−Trp/−Leu/−His/−Ade agar medium containing 20 μg mL^− 1^ X-α-Gal and 125 μg mL^− 1^ aureobasidin A (SD/−Trp/−Leu/−His/−Ade/X-a-Gal/AbA). Blue colonies were identified via PCR and sequencing. The sequences were aligned to the reference genome of *Gossypium hirsutum* [54] and annotated. The ORFs of *GhTT2*, *GhBTF3* and *GhRD21A*, which encoded potential interacting proteins, were cloned into the pGADT7 vector. Each recombinant construct was co-transformed into the yeast strain Y2HGold with the pGBKT7-*GhWRKY27* plasmid. The yeast transformation products were assayed on both SD/−Trp/−Leu and SD/−Trp/−Leu/−His/−Ade/X-a-Gal/AbA medium at 30 °C for 3–5 days. The pGBKT7-p53 and pGADT7-largeT plasmids were used as positive controls, and pGBKT7-laminC and pGADT7-largeT were used as negative controls.

### BiFC

To detect protein interactions in vivo, the ORFs of the *GhWRKY27*, *GhTT2*, *GhBTF3* and *GhRD21A* genes were individually cloned into the pSPYNE-35S and pSPYCE-35S vectors. Combinations of the plasmid constructs (pSPYNE + pSPYCE, pSPYNE-*GhWRKY27* + pSPYCE, pSPYNE + pSPYCE-*GhWRKY27*, pSPYNE-*GhTT2* + pSPYCE, pSPYNE + pSPYCE-*GhTT2*, pSPYNE-*GhWRKY27* + pSPYCE-*GhTT2*, pSPYNE-*GhTT2* + pSPYCE-*GhWRKY27*, pSPYNE-*GhBTF3* + pSPYCE, pSPYNE + pSPYCE-*GhBTF3*, pSPYNE-*GhWRKY27* + pSPYCE-*GhBTF3*, pSPYNE-*GhBTF3* + pSPYCE-*GhWRKY27*, pSPYNE-*GhRD21A* + pSPYCE, pSPYNE + pSPYCE-*GhRD21A*, pSPYNE-*GhWRKY27* + pSPYCE-*GhRD21A* and pSPYNE-*GhRD21A* + pSPYCE-*GhWRKY27*) were co-transformed into *Arabidopsis* protoplasts. The BiFC assay was performed according to previously described protocols [55, 56]. The fluorescent signal was detected using an Olympus FV 1000 confocal microscope.

### Production of the recombinant *GhWRKY27* protein and anti-*GhWRKY27* preparation

The ORF of *GhWRKY27* was cloned into the pET-28a(+) prokaryotic expression vector to generate the pET-28a(+)-*GhWRKY27* fusion plasmid. The plasmid that encodes the 6 × His-tagged fusion protein was transformed into the *Escherichia coli* strain BL21 (DE3). To obtain the fusion protein, BL21 (DE3) cells harbouring the pET-28a(+)-*GhWRKY27* construct were cultured in LB liquid medium and induced by 0.5 mM isopropyl-β-D-thiogalactoside (IPTG) at 28 °C. The induced cells were then sonicated until the solution became clear. The supernatant was collected via centrifugation, and the recombinant proteins were purified using a His-tag Protein Purification Kit (Beyotime). The purified proteins were employed to immunize mice for the preparation of an anti-*GhWRKY27* monoclonal antibody (Loyel, Zhengzhou).

### ChIP-seq

ChIP-seq was used to identify *GhWRKY27* protein binding sites at the genome-wide scale in vivo. The ChIP assay was conducted according to protocols described previously [57]. Approximately 5-g senescent cotyledon samples of Liao4086 were collected and treated with 1% formaldehyde to crosslink and fix the DNA-protein complexes in the cells. The cells were then lysed, and chromatin was broken via ultrasound into DNA fragments with sizes of approximately 100–500 bp. The anti-*GhWRKY27* antibody was employed to immunoprecipitate the DNA-*GhWRKY27* protein complexes. Beads were added, and the bead-bound complexes were collected. The complexes were subsequently eluted and reverse cross-linked, and the DNA fragments were purified for construction of the sequencing library. The ends of the purified DNA fragments were repaired and attached to adapter sequences using a Paired-End DNA Sample Prep Kit (Illumina). The DNA fragments were amplified via PCR, and a fragment size of 100–500 bp was selected. The qualified library was used for HiSeq 2500 sequencing (Novogene, Beijing). The adapter sequence and low-quality data were removed from the raw sequencing data. The obtained clean reads were aligned to the reference genome of *Gossypium hirsutum* [54] using Short Oligonucleotide Analysis Package (SOAP2) [58]. Model-based Analysis for ChIP-Seq (MACS) was employed to identify peak calls at the whole-genome scale [59]. Anti-lgG was used as a control.

### Y1H assay

Based on the ChIP-seq results, 8 potential downstream target genes encoding proteins such as aspartic protease in guard cell 1 (Gh_A07G1258, *GhASPG1*), WRKY TF 1 (Gh_A11G1391, *GhWRKY1*), ripening-related protein 2 (Gh_A12G0955, *GhRipen2–1*), gibberellin 20 oxidase 2 (Gh_D05G2274, *Gh20ox2*), cytochrome P450 94C1 (Gh_D08G0085, *GhCYP94C1)*, IAA-amido synthetase GH3.5 (Gh_D11G1006, *GhGH3.5*), auxin-induced protein 15A (Gh_D12G0291, *GhIAA15A*) and ripening-related protein 2 (Gh_D12G1102, *GhRipen2–2*) were verified using a Y1H system. Three copies of specific promoter fragments were cloned into the pHIS2 vector. The fragments came from the highly enriched promoter regions observed through ChIP-seq analysis. The fragment sequences are listed in Additional file 3: Table S3. The ORF of *GhWRKY27* was amplified and cloned into the pGADT7 vector. Each pHIS2 bait vector containing a target gene was co-transformed into the competent yeast strain Y187 with the pGADT7-*GhWRKY27* prey vector. The transformants were selected on SD/−Trp/−Leu medium, and the co-transformed cells were further identified on SD/−Trp/−Leu/−His medium containing 200 mM 3-amino-1,2,4-triazole (3-AT) (SD/−Trp/−Leu/−His + 200 mM 3-AT). The combination of pGADT7-*GhWRKY27* and pHIS2 was employed as a negative control plasmid.

### EMSA

For EMSA, the specific promoter fragments of *GhCYP94C1* and *GhRipen2–2* containing the W-box and mutated W-box were synthesized as biotin end-labelled and unlabelled oligonucleotides. The unlabelled W-box oligonucleotide served as a competitor. The anti-*GhWRKY27* antibody was employed for supershift identification. The assay was performed using the LightShift® Chemiluminescent EMSA Kit (Thermo Scientific, Waltham, MA, USA) according to the manufacturer’s instructions.

### Dual-luciferase reporter assay

Transient reporter expression was assayed in tobacco leaves using a dual-luciferase reporter system [60]. The *GhCYP94C1* and *GhRipen2–2* promoters were amplified and inserted into the pGreenII0800-LUC vector as the reporter plasmid. The ORF of *GhWRKY27* was amplified and inserted into the pGreenII 62-SK vector as the effecter plasmids. The fusion construct plasmids were transformed into *Agrobacterium tumefaciens* strain GV3101 (pSoup-p19). The GV3101 (pSoup-p19) cells containing the recombinant plasmids were incubated in LB liquid medium containing 50 mg L^− 1^ kanamycin, gentamycin and rifampin until the OD600 value reached 0.5–0.6. Subsequently, the culture was adjusted to an OD600 value of 0.2 with infiltration buffer (10 mM MgCl_2_, 10 mM MES and 100 μM acetosyringone). The culture suspensions were left for 2 h at room temperature. The constructed effecter and reporter suspensions were mixed in a 1:1 ratio and co-infiltrated into tobacco leaves. After 2 days of infiltration, LUC and REN luciferase activity was detected using a dual-luciferase® reporter assay system (Promega, USA) on a Glomax 20/20 Luminometer (Promega, USA) according to the manufacturer’s instructions. At least six independent replicates were performed.

## Additional files

Additional file 1: **Table S1.** Partial result from library screening by the Y2H assay (DOCX 23 kb)

Additional file 2: **Table S2.** Detailed information regarding the target genes of *GhWRKY27* identified by ChIP-seq (DOCX 84 kb)

Additional file 3: **Table S3.** Nucleic acid sequences of the potential target genes of *GhWRKY27* used for the Y1H system (DOCX 24 kb)

Additional file 4: **Fig. S1.** The expression levels of *GhCYP94C1* in different tissues (TIFF 2064 kb)

Additional file 5: **Fig. S2.** The expression levels of *GhCYP94C1* at different stages of flower bud differentiation from different maturing varieties. (TIFF 124 kb)

Additional file 6: **Table S4.** Primers used in this study (DOCX 40 kb)

## Abbreviations

ChIP-seq: chromatin immunoprecipitation followed by sequencing
EMSA: Electrophoretic mobility shift assay
JA: Jasmonic acid
ORF: Open reading frame
qRT-PCR: quantitative real-time PCR
SA: Salicylic acid
SAGs: Senescence-associated genes
TFs: Transcription factors
WT: Wild-type
