# Supplementary material for: The WRKY transcription factor GhWRKY27 coordinates the senescence regulatory pathway in upland cotton (Gossypium hirsutum L.)
Source: BMC Plant Biol. 2019 Mar 29;19:116. doi: 10.1186/s12870-019-1688-z (PMC6440019; doi:10.1186/s12870-019-1688-z)
Supplement: Supplementary file 1 — Table S1. Partial result from library screening by the Y2H assay (DOCX 23 kb) [file 12870_2019_1688_MOESM1_ESM.docx]

**Table S1. Partial result from library screening by the Y2H assay**

| **Gene ID** | | **Gene Name** | **Annotation** |
| --- | --- | --- | --- |
| Gh_A02G0358 | ETR2 | | Ethylene receptor 2 |
| Gh_A05G1162 | | SBT1.7 | Subtilisin-like protease SBT1.7 |
| Gh_A07G0140 | | TT2 | MYB domain protein 3, transcription factor TT2 |
| Gh_A08G2018 | | BTF3 | Transcription factor BTF3 |
| Gh_A09G2498 | | CYP736A12 | Cytochrome P450 CYP736A12 |
| Gh_A12G0570 | | PMADS1 | Floral homeotic protein PMADS 1 |
| Gh_D01G1044 | | RD21A | Cysteine proteinase RD21a |
| Gh_D02G1418 | | At1g03400 (ACOH4) | 1-aminocyclopropane-1-carboxylate oxidase homolog 4 |
| Gh_D05G0138 | | IAA29 | Auxin-responsive protein IAA29 |
| Gh_D08G0970 | | RFS6 | Probable galactinol--sucrose galactosyltransferase 6 |
| Gh_D10G1189 | | HSP70-7 | Heat shock 70 kDa protein 7, chloroplastic |
| Gh_D11G3296 | | HSP70 | Heat shock cognate 70 kDa protein |
