# Supplementary material for: The WRKY transcription factor GhWRKY27 coordinates the senescence regulatory pathway in upland cotton (Gossypium hirsutum L.)
Source: BMC Plant Biol. 2019 Mar 29;19:116. doi: 10.1186/s12870-019-1688-z (PMC6440019; doi:10.1186/s12870-019-1688-z)
Supplement: Supplementary file 2 — Table S2. Detailed information regarding the target genes of GhWRKY27 identified by ChIP-seq (DOCX 84 kb) [file 12870_2019_1688_MOESM2_ESM.docx]

**Table S2. Detailed information regarding the target genes of *GhWRKY27* identified by ChIP-seq**

| **Gene ID** | **Gene name** | **No. of W-boxes (as-1)^a^** | **Annotation** | **Chr** | **Peak start** | **Peak end** | **Fold_enrichment** | **Chr(region)** | **Start(region)** | **End(region)** | **Strand** |
| --- | --- | --- | --- | --- | --- | --- | --- | --- | --- | --- | --- |
| Gh_A01G0590 | ATML1 | 1 (1) | Homeobox-leucine zipper protein MERISTEM L1 | A01 | 10573013 | 10573465 | 31.09 | A01 | 10572257 | 10574256 | + |
| Gh_A01G0821 | ATL80 | 0 (5) | RING-H2 finger protein ATL80 | A01 | 18758879 | 18758945 | 24.51 | A01 | 18758900 | 18760899 | - |
| Gh_A02G0772 | tmem50 | 1 (8) | Transmembrane protein 50 homolog | A02 | 15153584 | 15153823 | 38.36 | A02 | 15152037 | 15154036 | - |
| Gh_A03G0148 | XPB1 | 1 (3) | DNA repair helicase XPB1 | A03 | 2276716 | 2276933 | 73.53 | A03 | 2276182 | 2278181 | + |
| Gh_A03G0830 | At1g48360 | 1 (9) | Fanconi-associated nuclease 1 homolog | A03 | 43539952 | 43540195 | 34.6 | A03 | 43539048 | 43541047 | + |
| Gh_A04G1373 | rps12-B | 1 (2) | 30S ribosomal protein S12-B, chloroplastic | scaffold1234_A04 | 177095 | 177176 | 104.17 | scaffold894_A04 | 175383 | 177382 | + |
| Gh_A05G0820 | DOF1.1 | 0 (1) | Dof zinc finger protein DOF1.1 | A05 | 8243491 | 8243543 | 51.15 | A05 | 8243482 | 8245481 | - |
| Gh_A05G1489 | At1g16860 | 0 | Uncharacterized membrane protein At1g16860 | A05 | 15280510 | 15280582 | 82.56 | A05 | 15280381 | 15282380 | + |
| Gh_A05G2853 | rpl14 | 1 (3) | 50S ribosomal protein L14, chloroplastic | A05 | 64442786 | 64442998 | 50.12 | A05 | 64442588 | 64444587 | + |
| Gh_A05G3347 | RLK1 | 1 (3) | G-type lectin S-receptor-like serine/threonine-protein kinase RLK1 | A05 | 87426616 | 87426791 | 39.22 | A05 | 87424792 | 87426791 | - |
| Gh_A05G3871 | SNRNP25 | 0 (1) | U11/U12 small nuclear ribonucleoprotein 25 kDa protein | scaffold1234_A05 | 80287 | 80489 | 51.15 | scaffold1234_A05 | 79097 | 81096 | + |
| Gh_A06G0805 | B3GALT12 | 3 (6) | Probable beta-1,3-galactosyltransferase 12 | A06 | 29714176 | 29714334 | 58.82 | A06 | 29712351 | 29714350 | - |
| Gh_A06G1000 | At1g71810 | 0 (3) | Uncharacterized aarF domain-containing protein kinase At1g71810, chloroplastic | A06 | 48414677 | 48414762 | 89.5 | A06 | 48414602 | 48416601 | + |
| Gh_A07G1258^b^ | ASPG1 | 0 (8) | Protein aspartic protease in guard cell 1 | A07 | 28944980 | 28945072 | 127.86 | A07 | 28944556 | 28946555 | + |
| Gh_A07G1449 | TAR4 | 0 (4) | Tryptophan aminotransferase-related protein 4 | A07 | 41233945 | 41233998 | 51.15 | A07 | 41232641 | 41234640 | + |
| Gh_A07G2244 | CRRSP56 | 0 (2) | Cysteine-rich repeat secretory protein 56 | scaffold1865_A07 | 21327 | 21503 | 63.93 | scaffold1865_A07 | 20018 | 22017 | - |
| Gh_A08G0485 | NA | 1 (9) | Nitrate reductase [NADH] | A08 | 6734392 | 6734574 | 56.02 | A08 | 6734447 | 6736446 | - |
| Gh_A08G0724 | CYCB3-1 | 0 (4) | Putative cyclin-B3-1 | A08 | 19779140 | 19779390 | 51.15 | A08 | 19777804 | 19779803 | - |
| Gh_A08G1052 | At1g52740 | 1 (3) | Probable histone H2A variant 3 | A08 | 72905406 | 72905514 | 107.84 | A08 | 72905345 | 72907344 | - |
| Gh_A08G1904 | emc2-a | 0 (5) | ER membrane protein complex subunit 2-A | A08 | 99755863 | 99756029 | 89.5 | A08 | 99755041 | 99757040 | - |
| Gh_A08G2374 | Os05g0277500 | 1 (4) | Germin-like protein 5-1 | scaffold1937_A08 | 85555 | 85791 | 38.36 | scaffold1937_A08 | 83691 | 85690 | - |
| Gh_A08G2394 | CYP76B6 | 0 (6) | Geraniol 8-hydroxylase | scaffold1942_A08 | 12115 | 12353 | 32.68 | scaffold1942_A08 | 10794 | 12793 | + |
| Gh_A09G1238 | NA | 1 (2) | Flavonol sulfotransferase-like | A09 | 64391416 | 64391518 | 58.82 | A09 | 64390704 | 64392703 | + |
| Gh_A09G1239 | NA | 0 (2) | Flavonol 3-sulfotransferase | A09 | 64391416 | 64391518 | 58.82 | A09 | 64391209 | 64393208 | + |
| Gh_A09G1250 | rplV | 0 (4) | 50S ribosomal protein L22 | A09 | 64602390 | 64602602 | 46.14 | A09 | 64601957 | 64603956 | + |
| Gh_A09G2465 | psbK | 0 (1) | Photosystem II reaction center protein K | scaffold2309_A09 | 17832 | 17885 | 30.17 | scaffold2309_A09 | 16278 | 18277 | + |
| Gh_A10G0788 | ndhJ | 1(2) | NAD(P)H-quinone oxidoreductase subunit J, chloroplastic | A10 | 16073889 | 16073959 | 15.59 | A10 | 16073853 | 16075852 | - |
| Gh_A10G1492 | eif3j | 1 (3) | Eukaryotic translation initiation factor 3 subunit J | A10 | 81758619 | 81758834 | 43.57 | A10 | 81757176 | 81759175 | - |
| Gh_A10G1637 | PUB44 | 4 (10) | U-box domain-containing protein 44 | A10 | 87685306 | 87685523 | 51.15 | A10 | 87684314 | 87686313 | - |
| Gh_A10G1820 | SCPL18 | 0 (3) | Serine carboxypeptidase-like 18 | A10 | 93584242 | 93584302 | 63.93 | A10 | 93582308 | 93584307 | - |
| Gh_A11G0835 | LEA14-A | 4 (7) | Late embryogenesis abundant protein Lea14-A | A11 | 8426399 | 8426455 | 39.22 | A11 | 8424991 | 8426990 | + |
| Gh_A11G1391^b^ | WRKY1 | 0 (3) | WRKY transcription factor 1 | A11 | 18157208 | 18157408 | 46.14 | A11 | 18155748 | 18157747 | - |
| Gh_A11G1427 | Os06g0358800 | 1 (1) | Ribonuclease 3-like protein 3 | A11 | 19248279 | 19248441 | 68.2 | A11 | 19246723 | 19248722 | + |
| Gh_A11G1772 | KRP7 | 1 (1) | Cyclin-dependent kinase inhibitor 7 | A11 | 30799036 | 30799244 | 51.15 | A11 | 30797740 | 30799739 | + |
| Gh_A12G0848 | CBG02625 | 1 (2) | Protein EFR3 homolog | A12 | 56327530 | 56327768 | 38.36 | A12 | 56326218 | 56328217 | - |
| Gh_A12G0955^b^ | Os02g0637000 (Ripen2-1) | 0 (3) | Putative ripening-related protein 2 | A12 | 60248876 | 60248930 | 26.14 | A12 | 60247522 | 60249521 | - |
| Gh_A13G1025 | CYP93B1 | 2 (5) | Licodione synthase | A13 | 58064016 | 58064215 | 39.22 | A13 | 58062616 | 58064615 | - |
| Gh_D01G1550 | lip | 1 (3) | Lipase | D01 | 47955498 | 47955554 | 51.15 | D01 | 47953568 | 47955567 | - |
| Gh_D02G0277 | GSO1 | 0 (3) | LRR receptor-like serine/threonine-protein kinase GSO1 | D02 | 3311691 | 3311915 | 38.36 | D02 | 3311045 | 3313044 | - |
| Gh_D02G0597 | ANX2 | 0 (3) | Receptor-like protein kinase ANXUR2 | D02 | 8098263 | 8098479 | 29.41 | D02 | 8097270 | 8099269 | - |
| Gh_D02G1652 | NA | 2 (4) | NA | D02 | 56958690 | 56958847 | 49.02 | D02 | 56958367 | 56960366 | + |
| Gh_D03G0991 | PABN1 | 1 (2) | Polyadenylate-binding protein 1 | D03 | 33935394 | 33935567 | 49.02 | D03 | 33934184 | 33936183 | + |
| Gh_D03G1155 | GSTU7 | 2 (8) | Glutathione S-transferase U7 | D03 | 38035795 | 38035985 | 58.82 | D03 | 38034964 | 38036963 | - |
| Gh_D03G1157 | spr0982 | 2 (3) | Alpha-monoglucosyldiacylglycerol synthase | D03 | 38043144 | 38043546 | 28.01 | D03 | 38042565 | 38044564 | + |
| Gh_D04G0045 | VPS24-1 | 1 (4) | Vacuolar protein sorting-associated protein 24 homolog 1 | D04 | 717904 | 718167 | 38.36 | D04 | 716826 | 718825 | - |
| Gh_D04G0542 | AIM32 | 1 (6) | Altered inheritance of mitochondria protein 32 | D04 | 9333516 | 9333779 | 49.02 | D04 | 9332052 | 9334051 | + |
| Gh_D05G1096 | PRK4 | 1 (4) | Pollen receptor-like kinase 4 | D05 | 9340201 | 9340257 | 39.22 | D05 | 9339841 | 9341840 | - |
| Gh_D05G1097 | CASBPX1 | 0 (2) | Cycloartenol synthase | D05 | 9340201 | 9340257 | 39.22 | D05 | 9339553 | 9341552 | + |
| Gh_D05G1879 | At1g75040 | 0 (1) | Pathogenesis-related protein 5 | D05 | 17180600 | 17180819 | 93.37 | D05 | 17179506 | 17181505 | - |
| Gh_D05G2274^b^ | 20ox2 | 1 (6) | Gibberellin 20 oxidase 2 | D05 | 21923578 | 21923788 | 29.41 | D05 | 21923263 | 21925262 | + |
| Gh_D05G2755 | PU1 | 0 (3) | Pullulanase 1, chloroplastic | D05 | 29859406 | 29859613 | 63.93 | D05 | 29858838 | 29860837 | + |
| Gh_D05G2771 | PDPK1 | 3 (5) | 3-phosphoinositide-dependent protein kinase 1 | D05 | 30247330 | 30247522 | 52.98 | D05 | 30245734 | 30247733 | - |
| Gh_D05G3341 | RABA1F | 0 | Ras-related protein RABA1f | D05 | 54106211 | 54106466 | 38.36 | D05 | 54105367 | 54107366 | - |
| Gh_D05G3795 | NA | 1 (3) | NA | scaffold4064_D05 | 27647 | 27851 | 49.02 | scaffold4064_D05 | 27430 | 29429 | + |
| Gh_D06G0884 | FBL15 | 1 (2) | F-box/LRR-repeat protein 15 | D06 | 16716855 | 16716907 | 32.68 | D06 | 16716691 | 16718690 | - |
| Gh_D06G1099 | NA | 1 (1) | NA | D06 | 24852676 | 24852746 | 42.63 | D06 | 24852029 | 24854028 | + |
| Gh_D06G1751 | At3g56180 | 1 (2) | Protein LURP-one-related 14 | D06 | 57072303 | 57072457 | 54.47 | D06 | 57071808 | 57073807 | - |
| Gh_D07G0753 | At5g37990 | 0 | Probable S-adenosylmethionine-dependent methyltransferase At5g37990 | D07 | 9231166 | 9231336 | 38.36 | D07 | 9229875 | 9231874 | + |
| Gh_D07G1826 | FER | 0 (4) | Receptor-like protein kinase FERONIA | D07 | 43967686 | 43967770 | 76.72 | D07 | 43967576 | 43969575 | + |
| Gh_D07G2226 | UVH3 | 2 (9) | DNA repair protein UVH3 | D07 | 53299736 | 53299916 | 63.93 | D07 | 53298926 | 53300925 | - |
| Gh_D08G0085^b^ | CYP94C1 | 1 (3) | Cytochrome P450 94C1 | D08 | 690159 | 690299 | 62.39 | D08 | 689524 | 691523 | + |
| Gh_D08G0236 | NA | 0 | NA | D08 | 2260559 | 2260611 | 27.05 | D08 | 2259558 | 2261557 | - |
| Gh_D08G0830 | NA | 1 (5) | NA | D08 | 13877876 | 13877994 | 73.53 | D08 | 13877222 | 13879221 | + |
| Gh_D08G0861 | NA | 0 (1) | NA | D08 | 15487941 | 15487994 | 89.5 | D08 | 15487887 | 15489886 | - |
| Gh_D08G1306 | PPAN | 1 (3) | Peter Pan-like protein | D08 | 42994933 | 42995088 | 49.02 | D08 | 42993628 | 42995627 | - |
| Gh_D08G2118 | EDR2L | 1 (4) | Protein enhanced disease resistance 2-like | D08 | 60363157 | 60363208 | 37.35 | D08 | 60361460 | 60363459 | - |
| Gh_D08G2564 | TIFY10A | 0 (5) | Protein TIFY 10A | D08 | 65321727 | 65321793 | 35.01 | D08 | 65320805 | 65322804 | + |
| Gh_D08G2677 | NA | 2 (4) | NA | scaffold4250_D08 | 16774 | 17020 | 51.15 | scaffold4250_D08 | 16584 | 18583 | + |
| Gh_D09G0625 | NA | 2 (3) | Iridoid synthase | D09 | 29192620 | 29192809 | 68.2 | D09 | 29191508 | 29193507 | + |
| Gh_D09G0626 | VEP1 | 1 (3) | 3-oxo-Delta(4,5)-steroid 5-beta-reductase | D09 | 29192620 | 29192809 | 68.2 | D09 | 29192308 | 29194307 | + |
| Gh_D09G0682 | CRK29 | 1 (4) | Cysteine-rich receptor-like protein kinase 29 | D09 | 30613503 | 30613785 | 49.02 | D09 | 30613099 | 30615098 | + |
| Gh_D09G0685 | B120 | 0 (3) | G-type lectin S-receptor-like serine/threonine-protein kinase B120 | D09 | 30695631 | 30695700 | 52.29 | D09 | 30694022 | 30696021 | - |
| Gh_D09G1386 | NA | 0 (1) | Phosphoglycerate kinase, chloroplastic (Fragment) | D09 | 41361129 | 41361210 | 29.41 | D09 | 41360197 | 41362196 | + |
| Gh_D09G1870 | NA | 1 (3) | NA | D09 | 46223956 | 46224183 | 51.6 | D09 | 46223156 | 46225155 | + |
| Gh_D09G2121 | Dym | 0 (1) | Dymeclin | D09 | 48436981 | 48437033 | 41.28 | D09 | 48436227 | 48438226 | + |
| Gh_D10G0148 | NTF2 | 0 (5) | Nuclear transport factor 2 | D10 | 1195828 | 1196037 | 41.28 | D10 | 1194109 | 1196108 | - |
| Gh_D10G0149 | XTH8 | 1 (6) | Probable xyloglucan endotransglucosylase/hydrolase protein 8 | D10 | 1195828 | 1196037 | 41.28 | D10 | 1194982 | 1196981 | + |
| Gh_D10G0457 | HAK13 | 0 (3) | Probable potassium transporter 13 | D10 | 4317094 | 4317234 | 89.5 | D10 | 4315370 | 4317369 | + |
| Gh_D10G1417 | NA | 0 (1) | NA | D10 | 29850746 | 29850959 | 63.93 | D10 | 29850491 | 29852490 | + |
| Gh_D11G0119 | At5g52840 | 0 (7) | Probable NADH dehydrogenase [ubiquinone] 1 alpha subcomplex subunit 5, mitochondrial | D11 | 1135887 | 1136053 | 76.72 | D11 | 1135276 | 1137275 | + |
| Gh_D11G1006^b^ | GH3.5 | 2 (5) | Probable indole-3-acetic acid-amido synthetase GH3.5 | D11 | 8822553 | 8822622 | 115.08 | D11 | 8822243 | 8824242 | + |
| Gh_D11G1280 | RRF | 2 (4) | Ribosome-recycling factor, chloroplastic | D11 | 12208561 | 12208855 | 51.15 | D11 | 12207704 | 12209703 | + |
| Gh_D11G1488 | FH1 | 1 (4) | Formin-like protein 1 | D11 | 14839622 | 14839814 | 49.02 | D11 | 14838155 | 14840154 | - |
| Gh_D11G1939 | SBT1.7 | 3 (7) | Subtilisin-like protease SBT1.7 | D11 | 24204229 | 24204293 | 63.93 | D11 | 24203390 | 24205389 | - |
| Gh_D11G1957 | DEGP7 | 0 (2) | Protease Do-like 7 | D11 | 24926709 | 24926908 | 39.22 | D11 | 24925257 | 24927256 | - |
| Gh_D11G2557 | Os09g0297000 | 1 (3) | Ferrochelatase-1, chloroplastic | D11 | 52839940 | 52840182 | 63.93 | D11 | 52839403 | 52841402 | + |
| Gh_D11G2704 | CDL1 | 1 (7) | Serine/threonine-protein kinase CDL1 | D11 | 56376142 | 56376195 | 42.63 | D11 | 56375220 | 56377219 | - |
| Gh_D12G0103 | wdr26 | 1 (3) | WD repeat-containing protein 26 | D12 | 1327380 | 1327623 | 51.15 | D12 | 1326500 | 1328499 | + |
| Gh_D12G0123 | GLR3.2 | 2 (4) | Glutamate receptor 3.2 | D12 | 1614294 | 1614379 | 58.82 | D12 | 1613624 | 1615623 | - |
| Gh_D12G0291^b^ | IAA15A | 1 (4) | Auxin-induced protein 15A | D12 | 3981258 | 3981490 | 38.36 | D12 | 3980683 | 3982682 | - |
| Gh_D12G1022 | NA | 1 (4) | Probable serine/threonine-protein kinase | D12 | 35976910 | 35977087 | 49.02 | D12 | 35975504 | 35977503 | - |
| Gh_D12G1023 | slr0305 | 1 (3) | TVP38/TMEM64 family membrane protein slr0305 | D12 | 35976910 | 35977087 | 49.02 | D12 | 35976576 | 35978575 | + |
| Gh_D12G1102^b^ | Os02g0637000 (Ripen2-2) | 2 (7) | Putative ripening-related protein 2 | D12 | 37513737 | 37513911 | 37.35 | D12 | 37512325 | 37514324 | + |
| Gh_D13G0444 | P4H9 | 3 (3) | Probable prolyl 4-hydroxylase 9 | D13 | 5160308 | 5160524 | 39.22 | D13 | 5160392 | 5162391 | + |
| Gh_D13G2094 | COV1 | 3 (6) | Protein continuous vascular ring 1 | D13 | 56415835 | 56415897 | 68.63 | D13 | 56413878 | 56415877 | + |
| Gh_Sca004815G01 | NA | 0 (4) | NA | scaffold4815 | 24918 | 25061 | 63.93 | scaffold4815 | 23392 | 25391 | + |
| Gh_Sca004815G02 | NA | 1 (2) | NA | scaffold4815 | 34230 | 34289 | 32.68 | scaffold4815 | 32764 | 34763 | + |
| Gh_Sca007622G01 | psbE | 1 (1) | Cytochrome b559 subunit alpha | scaffold7622 | 789 | 843 | 49.02 | scaffold7622 | 0 | 1247 | + |

^a^ W-box: 5’-TTGACC/T-3’; as-1 box: 5’-TGAC-3’

^b^ Confirmed by yeast one-hybrid
