# Supplementary material for: The WRKY transcription factor GhWRKY27 coordinates the senescence regulatory pathway in upland cotton (Gossypium hirsutum L.)
Source: BMC Plant Biol. 2019 Mar 29;19:116. doi: 10.1186/s12870-019-1688-z (PMC6440019; doi:10.1186/s12870-019-1688-z)
Supplement: Supplementary file 3 — Table S3. Nucleic acid sequences of the potential target genes of GhWRKY27 used for the Y1H system (DOCX 24 kb) [file 12870_2019_1688_MOESM3_ESM.docx]

**Table S3. Nucleic acid sequences of the potential target genes of *GhWRKY27* used for the Y1H system**

| **Gene ID** | **Gene Name** | **Sequence** |
| --- | --- | --- |
| Gh_A07G1258 | ASPG1 | AGTTGGGACTGAGGAGAAAAATGGCAGCAGCATCGTCATCAGCAAGGACTACTGAGTTGGGACTGAGGAGAAAAATGGCAGCAGCATCGTCATCAGCAAGGACTACTGAGTTGGGACTGAGGAGAAAAATGGCAGCAGCATCGTCATCAGCAAGGACTACTG |
| Gh_A11G1391 | WRKY1 | GTTCGACCATCAACTTCTATCCGTGACATCAATTCTTTGGTCCGAGTTCGACCATCAACTTCTATCCGTGACATCAATTCTTTGGTCCGAGTTCGACCATCAACTTCTATCCGTGACATCAATTCTTTGGTCCGA |
| Gh_A12G0955 | Ripen2-1 | TAGTCCTTTGTCCAGGAATATTTTATATTTAGTCCATTTTATTTTACGCGCGTTTTAGTCCTTTGTCCAGGAATATTTTATATTTAGTCCATTTTATTTTACGCGCGTTTTAGTCCTTTGTCCAGGAATATTTTATATTTAGTCCATTTTATTTTACGCGCGTTT |
| Gh_D05G2274 | 20ox2 | ATAGGAACTAAAAATGACCAAATTAAAGTATATAGACCAACCCACCACCACCAGTCATAACCCCTTCTAGCACATAGGAACTAAAAATGACCAAATTAAAGTATATAGACCAACCCACCACCACCAGTCATAACCCCTTCTAGCACATAGGAACTAAAAATGACCAAATTAAAGTATATAGACCAACCCACCACCACCAGTCATAACCCCTTCTAGCAC |
| Gh_D08G0085 | CYP94C1 | CCAGCTCCTCGCGAAAAGAAAAATGACTAAAAAAATATTTAATTCTTAGTCCAGCTCCTCGCGAAAAGAAAAATGACTAAAAAAATATTTAATTCTTAGTCCAGCTCCTCGCGAAAAGAAAAATGACTAAAAAAATATTTAATTCTTAGT |
| Gh_D11G1006 | GH3.5 | AGGAGCATATTAACGTGTATTGTGATAGTCAAAGTGCTATTCATTTAGCCAAGAATCAAAGGAGCATATTAACGTGTATTGTGATAGTCAAAGTGCTATTCATTTAGCCAAGAATCAAAGGAGCATATTAACGTGTATTGTGATAGTCAAAGTGCTATTCATTTAGCCAAGAATCAA |
| Gh_D12G0291 | IAA15A | CTAAGTTGATTACGATCAAAGTCAAAGATTGAAGAAGAAAGTTGTTTGAACTAAGTTGATTACGATCAAAGTCAAAGATTGAAGAAGAAAGTTGTTTGAACTAAGTTGATTACGATCAAAGTCAAAGATTGAAGAAGAAAGTTGTTTGAA |
| Gh_D12G1102 | Ripen2-2 | CTGGGCTCAGGTCAGAAAAGCTTTACTCGGGGCTTGACTCGTTTTCTAACTGGGCTCAGGTCAGAAAAGCTTTACTCGGGGCTTGACTCGTTTTCTAACTGGGCTCAGGTCAGAAAAGCTTTACTCGGGGCTTGACTCGTTTTCTAA |
