# Supplementary figures and images for: The WRKY transcription factor GhWRKY27 coordinates the senescence regulatory pathway in upland cotton (Gossypium hirsutum L.)
Source: BMC Plant Biol. 2019 Mar 29;19:116. doi: 10.1186/s12870-019-1688-z (PMC6440019; doi:10.1186/s12870-019-1688-z)

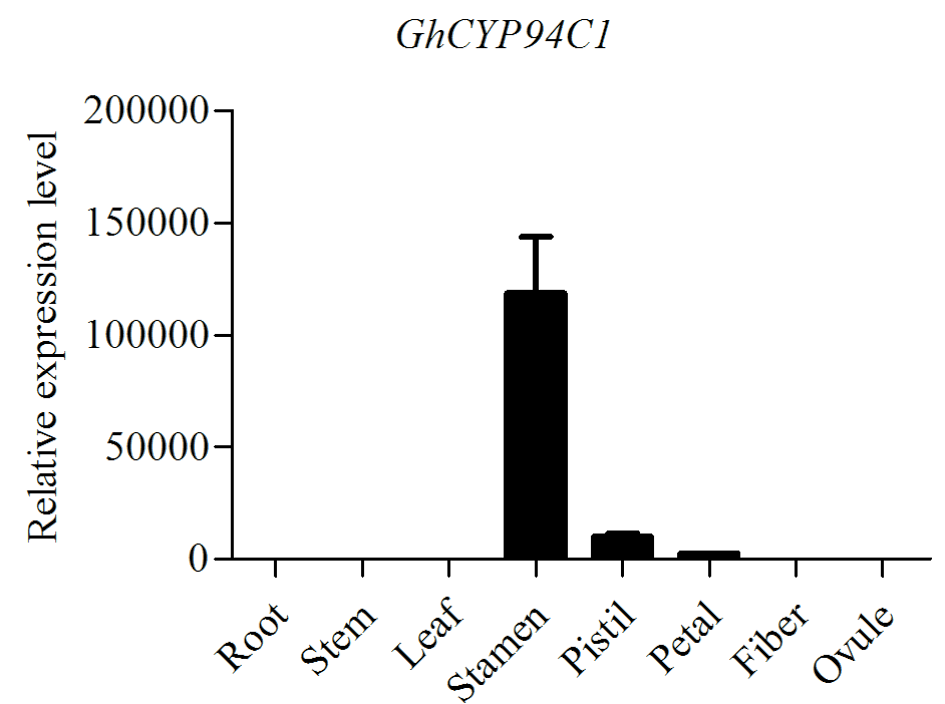

Supplement: Supplementary file 4 — Fig. S1. The expression levels of GhCYP94C1 in different tissues (TIFF 2064 kb) [file 12870_2019_1688_MOESM4_ESM.tiff]

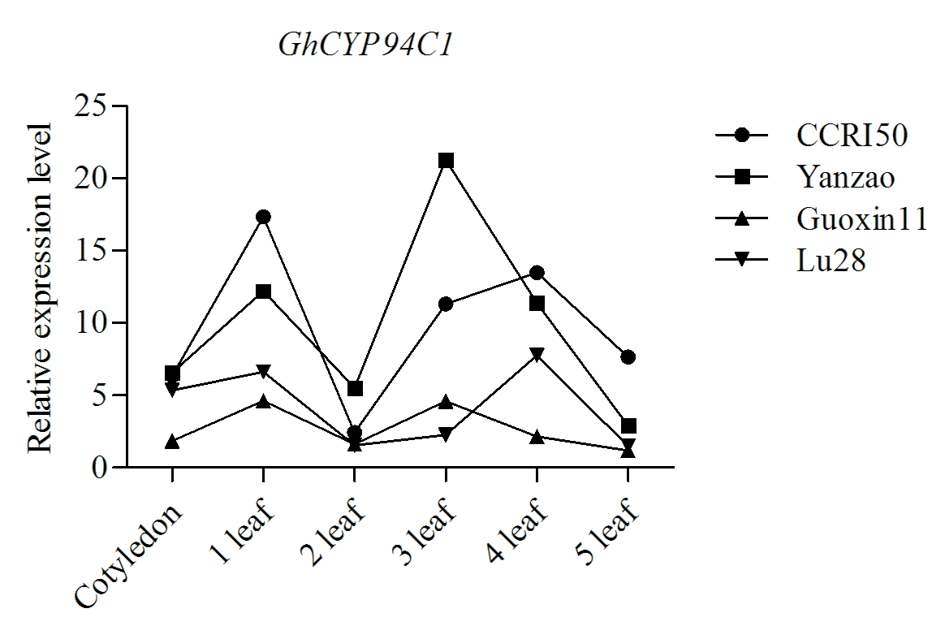

Supplement: Supplementary file 5 — Fig. S2. The expression levels of GhCYP94C1 at different stages of flower bud differentiation from different maturing varieties. (TIFF 124 kb) [file 12870_2019_1688_MOESM5_ESM.tiff]
