# Supplementary material for: The WRKY transcription factor GhWRKY27 coordinates the senescence regulatory pathway in upland cotton (Gossypium hirsutum L.)
Source: BMC Plant Biol. 2019 Mar 29;19:116. doi: 10.1186/s12870-019-1688-z (PMC6440019; doi:10.1186/s12870-019-1688-z)
Supplement: Supplementary file 6 — Table S4. Primers used in this study (DOCX 40 kb) [file 12870_2019_1688_MOESM6_ESM.docx]

**Table S4. Primers used in this study**

| **Primer name** | **Sequence ( 5’- 3’)** |
| --- | --- |
| gene clone-*WRKY27*-F | ATGGAGAACATGTGGAAGTGGG |
| gene clone-*WRKY27*-R | TTAGGAGAAAAATCCCGGTGT |
| promoter-*WRKY27*-F | ATATTATATTTTTAATGAGTA |
| promoter-*WRKY27*-R | ATGGAGAACATGTGGAAGTGGG |
| qRT-PCR-*GhActin*-F | ATCCTCCGTCTTGACCTTG |
| qRT-PCR-*GhActin*-R | TGTCCGTCAGGCAACTCAT |
| qRT-PCR-*WRKY27*-F | CCTACGGAACAGCCACAACAA |
| qRT-PCR-*WRKY27*-R | CTTCCAGCAGGTTTTCAGAGC |
| qRT-PCR-*TT2*-F | ATCCCGGAGTTGTATCGAACC |
| qRT-PCR-*TT2*-R | CCATGACTGTACTCCCCGTTG |
| qRT-PCR-*CYP94C1*-F | CTCCTTCATCTTCTTCACTTTCACG |
| qRT-PCR- *CYP94C1*-R | TGCCGAGGATATGAATATGGATTGT |
| qRT-PCR-*Ripen2-2*-F | GTGTCGAGCCATACAAAAGCAA |
| qRT-PCR- *Ripen2-2*-R | CATCATCCGAGTGGTAATGGTTGTC |
| qRT-PCR-*AtUBQ10*-F | AGATCCAGGACAAGGAAGGTATTC |
| qRT-PCR-*AtUBQ10*-R | CGCAGGACCAAGTGAAGAGTAG |
| qRT-PCR-*AtNAP*-F | GCCATTCACAGCGGTTCAAG |
| qRT-PCR-*AtNAP*-R | CAACAAATGAGCCAGCGAAC |
| qRT-PCR-*AtORE1*-F | CTTACCATGGAAGGCTAAGATGGG |
| qRT-PCR-*AtORE1*-R | TTCCAATAACCGGCTTCTGTCG |
| qRT-PCR-*AtSAG12*-F | TCCAATTCTATTCGTCTGGTGTGT |
| qRT-PCR-*AtSAG12*-R | CCACTTTCTCCCCATTTTGTTC |
| qRT-PCR-*AtSAG13*-F | GTGCCAGAGACGAAACTC |
| qRT-PCR-*AtSAG13*-R | GCTGTAAACTCTGTGGTC |
| Y2H-*WRKY27*-F(EcoRI) | CATGGAGGCCGAATTCATGGAGAACATGTGGAAGTG |
| Y2H-*WRKY27*-R (BamHI) | GCAGGTCGACGGATCCTTAGGAGAAAAATCCCGGTG |
| Y2H-*BTF3*-F(EcoRI) | GGAGGCCAGTGAATTCATGAACAAGGAGAGGCT |
| Y2H-*BTF3*-R (BamHI) | CGAGCTCGATGGATCCCTATTTAGCAGCTTGGCC |
| Y2H- *TT2*-F(EcoRI) | GGAGGCCAGTGAATTCATGGGGAGGAGGCCTT |
| Y2H- *TT2*-R (BamHI) | CGAGCTCGATGGATCCTTAGTTGATCCATTCATCTTCA |
| Y2H- *RD21A*-F(EcoRI) | GGAGGCCAGTGAATTCATGGGTTCTCAGGGATC |
| Y2H- *RD21A*-R (BamHI) | CGAGCTCGATGGATCCTCAATGTGCCCAGAACG |
| BiFC-*WRKY27*-F (XbaI) | CTAGTCTAGAATGGAGAACATGTGGAAGTG |
| BiFC-*WRKY27*-R (BamHI) | CGCGGATCCTTAGGAGAAAAATCCCGGTG |
| BiFC-*RD21A*-F (XbaI) | CTAGTCTAGAATGGGTTCTCAGGGATC |
| BiFC-*RD21A*-R (BamHI) | CGCGGATCCTCAATGTGCCCAGAACG |
| BiFC-*TT2*-F (BamHI) | CGCGGATCCATGGGGAGGAGGCCTT |
| BiFC-*TT2*-R (XhoI) | CCGCTCGAGTTAGTTGATCCATTCATCTTCA |
| BiFC-*BTF3*-F (XbaI) | CTAGTCTAGAATGAACAAGGAGAGGCT |
| BiFC-*BTF3*-R (BamHI) | CGCGGATCCCTATTTAGCAGCTTGGCC |
| Y1H-*WRKY27*-F(EcoRI) | GGAGGCCAGTGAATTCATGGAGAACATGTGGAAGTG |
| Y1H-*WRKY27*-R(BamHI) | CGAGCTCGATGGATCCTTAGGAGAAAAATCCCGGTG |
| pET-28a(+)-*WRKY27*-F(BamHI) | ATGGGTCGCGGATCCATGGAGAACATGTGGAAGTG |
| pET-28a(+)-*WRKY27*-R(HindIII) | TGCGGCCGCAAGCTTTTAGGAGAAAAATCCCGGTG |
| SK-*WRKY27*-F(BamHI) | AGAACTAGTGGATCCATGGAGAACATGTGGAAGTG |
| SK-*WRKY27*-R(XhoI) | GGGCCCCCCCTCGAGTTAGGAGAAAAATCCCGGTG |
| LUC-*CYP94C1*-F(XhoI) | GGGCCCCCCCTCGAGACTACCCAATACTCAGTAGATC |
| LUC-*CYP94C1*-R(BamHI) | AGAACTAGTGGATCCTTTGATTGATGGAAAGAAAATAAG |
| LUC-*Ripen2-2*-F(XhoI) | GGGCCCCCCCTCGAGGGACCAAATAAAAATGCTAAAAC |
| LUC-*Ripen2-2*-R(BamHI) | AGAACTAGTGGATCCCTTGAATTTCTTTTGTTAAATCTTT |
| M13(-47) | TAATACGACTCACTATAGGG |
| 35S | GACGCACAATCCCACTATCC |
| T7 | TAATACGACTCACTATAGGG |
| 3’AD-R | AGATGGTGCACGATGCACAG |
| 3’BD-R | AAGAGTCACTTTAAAATTTGTAT |
| pHIS2-R | TGCCTGCTCATTTTTTAGTATA |
